# When flexibility feels like control: competing autonomy and Technostrain pathways linking digital work flexibility to employee well-being

**DOI:** 10.3389/fpsyg.2026.1977600

**Published:** 2026-10-22

**Authors:** Weina Liao

**Affiliations:** School of Economics and Management, Nanchang University, Nanchang, China

**Keywords:** after-hours availability expectations, autonomy paradox, digital autonomy, digital work flexibility, employee well-being, Technostrain

## Abstract

Digital work flexibility may be accompanied by both greater discretion and greater technology-related strain. We examine whether Digital Work Flexibility is associated with Digital Autonomy and Technostrain, how these two experiences are associated with Employee Well-Being, and whether employees’ perceived After-hours Availability Expectations (AAE) condition the two first-stage relationships. Survey data from 462 full-time employees in Jiangxi Province, China, with one respondent per organization, were analyzed using PLS-SEM. Digital Work Flexibility was positively associated with both Digital Autonomy and Technostrain. Digital Autonomy was positively associated with Employee Well-Being, whereas Technostrain was negatively associated with it. The two indirect relationships were significant and opposite in direction, and their relative balance varied across levels of perceived AAE: the flexibility-autonomy association was weaker and the flexibility-technostrain association stronger at higher AAE. These findings are consistent with a conditional resource-strain pattern in digitally flexible work. Because the data are cross-sectional and self-reported, the indirect relationships are interpreted as statistical associations rather than established causal mechanisms.

## 1 Introduction

Digital transformation research spans multiple levels of analysis. At the organizational level, digital innovation has been examined as part of enterprise-management transformation, including implications for managerial efficiency and resource utilization (Wang and Lee, 2023). Related firm-level research on new start-up SMEs links coopetition with access to external resources, organizational capabilities, and core competency under environmental turbulence (Wang and Wu, 2025). The present study focuses instead on employee-level work design. Digital technologies have loosened many of the temporal and spatial limits that once defined where work could be done and when it should end (Allen et al., 2015; Kossek et al., 2023; Harrop et al., 2026). Remote, hybrid, and other digitally enabled arrangements give employees more opportunities to decide where and when work is carried out (Charalampous et al., 2019; Shifrin and Michel, 2022; Harrop et al., 2026). However, the same arrangements may also extend work beyond traditional temporal boundaries because employees can remain reachable through digital communication channels (Allen et al., 2021; Nilsen et al., 2026). Thus, flexible digital work may be experienced differently depending on whether employees report greater discretion or stronger expectations to remain connected after hours (Scholze and Hecker, 2024).

In this study, Digital Work Flexibility is examined in a specific sense: employees’ perceived discretion regarding when and where work is performed in digitally enabled settings (Jones et al., 2008). This definition focuses on temporal and spatial flexibility rather than autonomy over work decisions or methods. Such a distinction follows prior work that separates flexibility dimensions according to the type of discretion employees exercise (Allen et al., 2013; Kossek et al., 2023; Harrop et al., 2026). The term “digital” identifies the work setting rather than a separate dimension of the construct (Parker and Grote, 2022).

Evidence on digitally enabled work does not point in a single direction. Flexible arrangements are often associated with greater choice and a stronger sense of control (Shifrin and Michel, 2022; Baum and Rau, 2024). At the same time, digital boundary crossing is also associated with persistent connectivity, information processing, and technology-related demands (Marsh et al., 2024; Kotek and Vranjes, 2025). Anxiety, fatigue, scepticism, and inefficacy in this setting are captured here as Technostrain (Salanova et al., 2013). Reading these streams together raises a more useful question than whether flexibility is simply beneficial or harmful: can the same work arrangement be associated with both a resource-related experience and a strain-related experience, and how are those experiences related to well-being?

A second issue concerns the boundary between flexibility and autonomy. Digital Work Flexibility concerns discretion over when and where work is performed. Digital Autonomy, by contrast, refers to experienced control over work decisions and work methods in digitally mediated work (Morgeson and Humphrey, 2006). Here, “digital” locates autonomy in a technology-intensive work setting; it does not redefine autonomy or imply control over platforms, algorithms, or devices (Parker and Grote, 2022). Greater flexibility does not necessarily coincide with greater decision or method autonomy (Baum and Rau, 2024), and broader work boundaries may also coincide with more exposure to digital demands and Technostrain (Becker and Lanzl, 2023; Kotek and Vranjes, 2025).

One feature of the digital-work context that may be especially relevant is the expectation of remaining available after normal working hours. After-hours Availability Expectations (AAE) refer to employees’ perceptions that they are expected to remain reachable beyond regular working time (Derks et al., 2015; Piszczek, 2017). Such expectations have been linked to poorer detachment and more persistent after-hours work connectivity (Mellner, 2016; Barber et al., 2019; He et al., 2024; Nilsen et al., 2026). AAE may therefore be relevant to whether flexible work is accompanied more strongly by autonomy-related or strain-related experiences.

Against this background, the present study examines two parallel indirect relationships between Digital Work Flexibility and Employee Well-Being, through Digital Autonomy and Technostrain, and whether the first-stage associations vary across levels of employees’ perceived AAE (Piszczek, 2017; Nilsen et al., 2026). The incremental contribution is deliberately specific. Rather than proposing that digital flexibility is uniquely beneficial or harmful, the study distinguishes when/where flexibility from decision/method autonomy and tests whether perceived after-hours availability expectations are associated with a different balance between an autonomy-related pathway and a technostrain-related pathway. Considering these pathways together also helps distinguish a weak overall DWF-EWB association from the coexistence of two statistically meaningful indirect relationships with opposite signs.

## 2 Theory and hypotheses development

### 2.1 Theoretical foundations: JD-R theory and the autonomy paradox

Digital work flexibility presents a theoretical challenge because the same work arrangement may be associated with different employee experiences. Digitally enabled flexibility can broaden discretion over when and where work is performed, while the same work context can also involve communication intensity, information processing, and expectations of continued accessibility. A useful explanation therefore needs to accommodate the coexistence of resource-related and strain-related experiences without assuming that one necessarily causes the other in cross-sectional data.

Job Demands-Resources (JD-R) Theory provides the first foundation for this distinction. The theory separates job resources that support goal achievement and employee functioning from job demands that require sustained effort and are associated with strain when excessive (Bakker and Demerouti, 2017; Bakker et al., 2023; Bakker and Demerouti, 2024; Demerouti, 2025). Applied to digitally enabled work, JD-R Theory offers a basis for examining Digital Autonomy as a resource-related experience and Technostrain as a strain-related experience. Importantly, Digital Work Flexibility itself is not treated here as an inherently beneficial resource; rather, the model asks whether reported flexibility is associated with both forms of experience in parallel (Parker and Grote, 2022; Marsh et al., 2024; Kotek and Vranjes, 2025).

The Autonomy Paradox provides a complementary, more contextual perspective. Prior work shows that mobile and digitally mediated work can coexist with greater discretion and stronger expectations of accessibility (Mazmanian et al., 2013). Research on telepressure, after-hours communication, and boundary management likewise documents tension between flexibility and perceived pressure to remain responsive outside normal working time (Barber and Santuzzi, 2015; Mellner, 2016; Barber et al., 2019; He et al., 2024). In the present study, the Autonomy Paradox is used as a theoretical lens for this tension rather than as a construct that is fully operationalized by the Digital Autonomy measure.

Together, the two perspectives have distinct roles in the model. JD-R Theory explains why resource-related and strain-related experiences can be examined simultaneously, whereas the Autonomy Paradox motivates attention to the surrounding connectivity context. Accordingly, employees’ perceived After-hours Availability Expectations (AAE) are examined as a boundary condition for the associations of Digital Work Flexibility with Digital Autonomy and Technostrain. This theoretical framing does not imply that the cross-sectional design establishes the proposed psychological sequence; it provides a basis for testing whether the observed conditional associations are consistent with the proposed resource-strain account.

### 2.2 Digital work flexibility and the two psychological pathways

Digital Work Flexibility concerns the latitude employees have over when and where they work in digitally enabled work settings (Jones et al., 2008). Temporal and spatial discretion remain central in current flexible-work research (Kossek et al., 2023; Harrop et al., 2026). The ability to work beyond a conventional office or schedule does not, by itself, indicate autonomy over how work is done. Accordingly, the construct is limited to when and where employees can work.

Digital Autonomy captures a different kind of discretion: employees’ experienced control over work decisions and work methods in digitally mediated work (Morgeson and Humphrey, 2006). The label identifies the context in which autonomy is exercised, not a new autonomy domain, and it does not refer to control over digital technologies themselves (Parker and Grote, 2022). Nor does this measure operationalize scheduling or boundary-control autonomy. Those domains are kept separate because temporal discretion is already part of Digital Work Flexibility.

The distinction can be stated simply. Flexibility concerns when and where work is done; autonomy concerns how employees carry out work and make work-related decisions (Jones et al., 2008; Morgeson and Humphrey, 2006). Even so, the two may be positively related. When flexible arrangements leave more room for adjustment, employees may also report more opportunities to use their own judgment and choose work methods (Wang et al., 2021; Baum and Rau, 2024; Olivo et al., 2025).

*H1a*. Digital Work Flexibility is positively associated with Digital Autonomy.

Because Digital Work Flexibility and after-hours availability both extend beyond conventional work boundaries, Employee Well-Being is examined broadly across life, workplace, and psychological domains (Allen et al., 2021; Derks et al., 2015; Zheng et al., 2015).

Digital Autonomy represents a work resource because it reflects room for judgment over decisions and methods (Bakker et al., 2023). In digitally mediated work, employees may still differ considerably in how much discretion they experience even when they use similar technologies. Greater decision and method autonomy has been associated with more favorable well-being in flexible and technology-intensive work settings (Wan et al., 2024; Olivo et al., 2025). A positive association between Digital Autonomy and Employee Well-Being is expected.

*H2a*. Digital Autonomy is positively associated with Employee Well-Being.

The same flexibility can also coincide with wider exposure to digital work. When temporal and spatial boundaries are less fixed, employees may encounter messages, online tasks, and changing technologies across a broader range of times and settings (Becker and Lanzl, 2023; Marsh et al., 2024). Such conditions are theoretically relevant to Technostrain because anxiety, mental fatigue, scepticism, and perceived inefficacy are psychological reactions reported in technology-intensive work (Salanova et al., 2013). The present study does not directly measure message volume, response speed, digital workload, or actual after-hours technology use; persistent connectivity is therefore treated as a plausible theoretical explanation rather than a mechanism directly observed in the data. Recent evidence nevertheless reports associations between flexible digital-work conditions and technology-related strain (Simon et al., 2024; Kotek and Vranjes, 2025).

*H1b*. Digital Work Flexibility is positively associated with Technostrain.

Technostrain concerns employees’ adverse psychological reactions to digital work rather than the technology itself (Salanova et al., 2013). Anxiety and fatigue are associated with depleted psychological resources, while scepticism and inefficacy are tied to less favorable evaluations of work and competence. Reviews of technostress research consistently report negative associations with employee well-being and work-life outcomes (Bottaro et al., 2024; Kumar, 2024; Mansuroğlu and Smith, 2026). Accordingly, we expect a negative association with Employee Well-Being.

*H2b*. Technostrain is negatively associated with Employee Well-Being.

The two hypotheses point in opposite directions, which is intentional. Digital work can be associated with resource-related and strain-related experiences at the same time (Tarafdar et al., 2019; Scholze and Hecker, 2024). Digital Autonomy and Technostrain represent those two possibilities in the present model.

### 2.3 Competing indirect pathways of digital autonomy and Technostrain

Taken separately, the two first-stage relationships imply different associations with Employee Well-Being. Flexible work arrangements show heterogeneous employee outcomes across forms and work settings (Shifrin and Michel, 2022; Harrop et al., 2026). In this model, higher Digital Work Flexibility may coincide with higher Digital Autonomy and higher Technostrain, which are expected to be positively and negatively associated with well-being, respectively (Scholze and Hecker, 2024).

These pathways need not exclude one another. Digital technologies can be associated with favorable and unfavorable work experiences at the same time (Tarafdar et al., 2019; Parker and Grote, 2022). An employee may report greater discretion and still feel anxious, fatigued, or detached in relation to digital work. For that reason, the overall association between Digital Work Flexibility and Employee Well-Being may obscure two indirect relationships with opposite signs.

These opposing indirect relationships are consistent with the JD-R view that resource-related and strain-related experiences can coexist in digitally mediated work (Bakker et al., 2023; Scholze and Hecker, 2024).

*H3a*. Digital Work Flexibility has a significant positive indirect relationship with Employee Well-Being through Digital Autonomy.

*H3b*. Digital Work Flexibility has a significant negative indirect relationship with Employee Well-Being through Technostrain.

### 2.4 After-hours availability expectations as a boundary condition

Digital Work Flexibility concerns discretion over when and where work is performed, but this form of flexibility need not coincide with greater decision or method autonomy. One relevant contextual factor is perceived availability pressure. After-hours Availability Expectations (AAE) refer here specifically to employees’ perceptions that their supervisors expect them to remain reachable and responsive beyond normal working hours (Derks et al., 2015; Piszczek, 2017). AAE is therefore an individual-level perceptual construct, not a directly measured organization-level norm or a measure of employees’ actual after-hours connectivity.

When perceived AAE are relatively low, flexibility over work time and location may be more strongly associated with employees’ sense of discretion over decisions and methods. By contrast, high perceived AAE may coexist with weaker autonomy-related associations because formal flexibility can occur alongside expectations of continued responsiveness (Barber and Santuzzi, 2015; Barber et al., 2019). This interpretation is consistent with the Autonomy Paradox as a contextual lens, while recognizing that the Digital Autonomy measure itself captures decision and method discretion rather than scheduling or boundary control (Mazmanian et al., 2013).

*H4a*. After-hours Availability Expectations negatively moderate the positive relationship between Digital Work Flexibility and Digital Autonomy, such that the relationship is weaker when After-hours Availability Expectations are high.

Perceived after-hours expectations may also coincide with a stronger association between flexible digital work and Technostrain. Prior research links expectations of responsiveness with reduced detachment and greater technology-related demands outside regular working hours (He et al., 2024; Nilsen et al., 2026). These findings make it plausible that DWF and Technostrain will be more strongly associated when perceived AAE are high, although the present study does not directly observe the behavioral connectivity process itself (Salanova et al., 2013; Kotek and Vranjes, 2025; Mansuroğlu and Smith, 2026).

*H4b*. After-hours Availability Expectations positively moderate the relationship between Digital Work Flexibility and Technostrain, such that the positive relationship is stronger when After-hours Availability Expectations are high.

Taken together, these arguments suggest a conditional pattern in which the associations of Digital Work Flexibility with autonomy-related and strain-related experiences differ across levels of employees’ perceived AAE.

### 2.5 Conditional indirect relationships with employee well-being

At lower perceived AAE, the positive DWF-Digital Autonomy association is expected to be stronger; given the positive association expected between Digital Autonomy and Employee Well-Being, the corresponding positive indirect relationship should also be larger. As perceived AAE increase, this autonomy-related indirect relationship is expected to become smaller (Mazmanian et al., 2013; Barber et al., 2019).

*H5a*. After-hours Availability Expectations moderate the positive indirect relationship between Digital Work Flexibility and Employee Well-Being through Digital Autonomy, such that the relationship is weaker when After-hours Availability Expectations are high.

The Technostrain pathway is expected to show the reverse ordering. Prior work associates stronger perceived after-hours expectations with more persistent work-related connectivity and technology-related demands (He et al., 2024; Kotek and Vranjes, 2025; Nilsen et al., 2026). Accordingly, the negative indirect relationship through Technostrain is expected to be larger in magnitude at higher perceived AAE (Mansuroğlu and Smith, 2026).

*H5b*. After-hours Availability Expectations moderate the negative indirect relationship between Digital Work Flexibility and Employee Well-Being through Technostrain, such that the negative relationship is stronger when After-hours Availability Expectations are high.

The model anticipates different indirect patterns across availability contexts: a relatively stronger autonomy-related pathway at lower AAE and a relatively stronger technostrain-related pathway at higher AAE. Figure 1 summarizes the proposed relationships.

**Figure 1:**
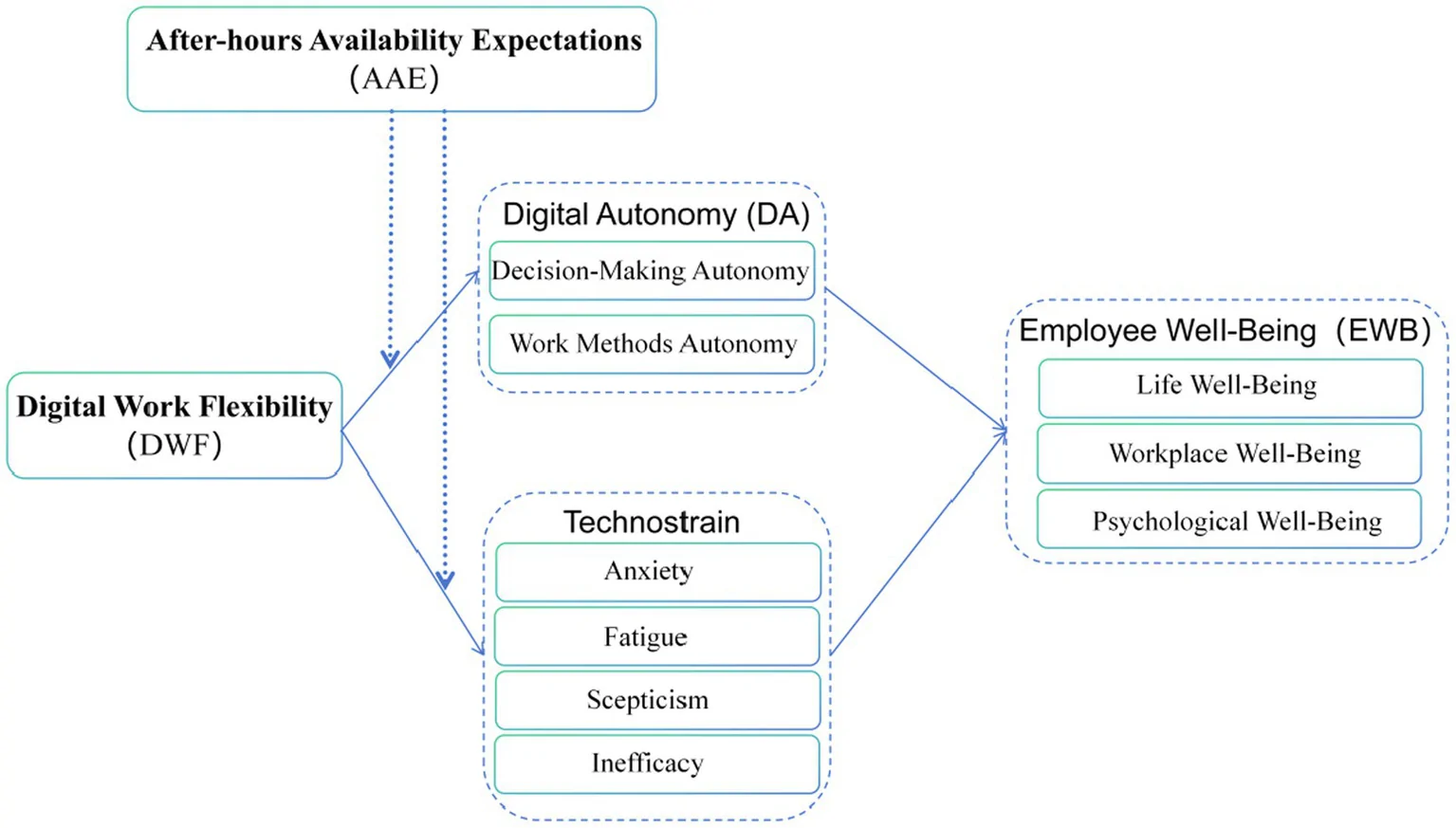
Research conceptual framework.

## 3 Materials and methods

### 3.1 Research design and sample

A cross-sectional survey was conducted among full-time employees in Jiangxi Province, China. Routine use of digital technologies for work tasks or communication was required for participation, and remote, hybrid, and on-site employees were eligible. Participants also had to have at least 6 months of tenure in their current organization and digital-work experience during the preceding 3 months. The target population was therefore full-time employees in Jiangxi who routinely engaged in digitally mediated work. Only one eligible employee was intended to be recruited from each participating organization.

Data collection ran from June to December 2025 through SOJUMP. The Jiangxi Association of Small and Medium Enterprises assisted in reaching eligible employees. Participation was voluntary. Before entering the questionnaire, respondents received information about the purpose of the study, anonymity and confidentiality, their right to withdraw, and informed consent. Ethical approval had been obtained from the relevant review body at Nanchang University (approval no. JXNCU20250619).

To reduce non-independence from multiple employees within the same organization, recruitment was restricted prospectively to one eligible focal employee per participating organization. The Jiangxi Association of Small and Medium Enterprises served as the organizational access point, while the research team provided the eligibility criteria and questionnaire instructions. One organization-linked invitation was allocated to each participating organization for forwarding to one eligible full-time employee. Organization-linked recruitment records, together with SOJUMP duplicate/device screening, were used to flag multiple or potentially non-independent submissions; if more than one eligible submission was linked to the same organization, only the first complete eligible response was retained. The final analytic dataset therefore contained 462 employees from 462 organizations. This one-respondent rule was a sampling and screening criterion at the individual level and does not imply that AAE or any other construct was measured at the organization level.

Sample size was assessed *a priori* in G*Power using the DWF × AAE interaction as the tested effect. The calculation specified *f*^2^ = 0.02, *α* = 0.05, power (1 − *β*) = 0.80, one tested predictor, and three predictors in the regression equation. This produced a minimum requirement of 395 respondents (Faul et al., 2009); the final analytic sample contained 462.

A total of 818 employees were invited, of whom 539 returned questionnaires, giving a response rate of 65.9%. Screening was applied sequentially and mutually exclusively, with each case assigned to the first criterion triggered. Fourteen questionnaires failed the eligibility criteria, 8 had substantial missing key data, 17 had unusually short completion times, 13 showed straight-lining or insufficient response variation, 16 failed attention checks, and 9 were duplicate or otherwise non-independent submissions. These 77 exclusions left 462 usable responses, representing 85.7% of returned questionnaires and 56.5% of all invitations. Supplementary Table S11 reports the full recruitment and screening flow.

Gender, age, education, organizational tenure, managerial level, weekly working hours, and primary work mode were recorded to describe the sample. Detailed industry and occupational-function categories were not retained in the analytic dataset; accordingly, the findings are generalized most cautiously to full-time employees using digital technologies who were reached through the regional SME-related recruitment network in Jiangxi Province. Control variables were included only where theoretically justified.

### 3.2 Measures and questionnaire development

The measurement approach followed the original definitions of the focal constructs. Adaptations were made only where the wording needed to reflect digitally mediated work, while the underlying meanings of the measures remained unchanged. Digital Work Flexibility, Digital Autonomy, After-hours Availability Expectations (AAE), and Employee Well-Being were measured using seven-point agreement scales (1 = strongly disagree, 7 = strongly agree). Technostrain was assessed using a seven-point frequency scale (1 = never, 7 = always or almost every day).

The English versions of the measures were translated into Chinese and independently back-translated before data collection. Three experts in organizational behavior, human resource management, and digital-work research reviewed the translated items. Their comments focused mainly on wording clarity and whether the items retained the intended meaning in a digital-work context. Particular attention was given to constructs with potentially close meanings, especially Digital Work Flexibility versus Digital Autonomy and AAE versus Technostrain. Minor wording refinements were made based on this process, while the original construct meanings were preserved (Brislin, 1970).

Digital Work Flexibility and Digital Autonomy were measured separately because they represent different forms of employee discretion. Digital Work Flexibility concerns employees’ perceived freedom regarding when and where work is performed and was assessed with the two-item operationalization reported by Jones et al. (2008). The source measure used two items covering perceived control/flexibility over when and where work was scheduled and used a four-point response scale. The present study retained both content domains, adapted the wording to digitally enabled work, and used the seven-point agreement format applied to the other focal perceptual constructs. The measure was not shortened statistically in the present study: one item refers to temporal flexibility and the other to spatial flexibility. The two items are combined because DWF is deliberately defined here as a parsimonious perception of when/where flexibility, consistent with the selected source operationalization; nevertheless, temporal and spatial flexibility may have distinct consequences, which we now acknowledge as a measurement boundary. Digital Autonomy refers to discretion over work decisions and methods and was measured using six items from the Work Design Questionnaire (Morgeson and Humphrey, 2006), including Decision-Making Autonomy and Work Methods Autonomy. Work Scheduling Autonomy was excluded not because scheduling control is theoretically unimportant, but because its timing content overlaps directly with the temporal component of DWF. Accordingly, Digital Autonomy reflects decision and method discretion rather than scheduling autonomy, boundary control, or control over digital systems.

Technostrain was measured using the 16-item scale validated by Salanova et al. (2013), comprising Anxiety, Fatigue, Scepticism, and Inefficacy. AAE was measured with four items adapted from the four-item supervisor-expectations measure reported by Derks et al. (2015). The source article describes the measure as supervisor expectations regarding smartphone use and staying connected to work during after-work hours and provides example items rather than reproducing all four questionnaire stems. We therefore describe the present items as adapted rather than as verbatim reproductions. The wording was aligned to after-hours digital communication while keeping the referent at employees’ perceptions of their supervisor’s expectations. The four adapted items cover free-time response, outside-hours reachability, prompt response during leisure time, and anticipated supervisor disapproval for nonresponse after hours. Thus, AAE represents employees’ perceived supervisor expectations rather than actual connectivity behavior or a shared organization-level norm. The translation/back-translation and expert-review procedure described above was applied to these adapted items. Supplementary Table S10 reports the scale sources, construct facets, item codes, adapted English wording, corresponding Chinese wording, and the source-to-adaptation information for DWF and AAE.

Employee Well-Being followed Zheng et al. (2015). The 18 items comprise six Life Well-Being, six Workplace Well-Being, and six Psychological Well-Being items. These three dimensions formed the higher-order Employee Well-Being construct, while separate dimension-level analyses were used to examine whether the structural results differed across the three domains.

A three-item Attitude Toward the Color Blue (ATCB) measure served as the marker variable because all focal constructs were self-reported. Its use follows Miller and Simmering (2023) and Simmering et al. (2015). Respondents rated “I prefer blue to other colors,” “I like the color blue,” and “I like blue clothes” on the seven-point agreement scale. ATCB was theoretically unrelated to the focal constructs and was used only in the assessment of potential common method variance. The full adapted item set is reported in Supplementary Table S10.

### 3.3 Data analysis strategy

SPSS was used for data screening and descriptive statistics. SmartPLS 4 was used to estimate the measurement and structural models with partial least squares structural equation modeling (PLS-SEM). PLS-SEM was selected because the analysis required a component-based framework for reflective-reflective higher-order constructs estimated with a two-stage approach, latent interaction terms, and bootstrapped conditional indirect relationships, while retaining an emphasis on explained variance in the endogenous constructs (Hair et al., 2019; Hair et al., 2022). The choice does not imply that the model was exploratory or that covariance-based SEM would be incapable of estimating the theoretical relationships; the structural paths were theory-driven and specified through *a priori* hypotheses.

Because the study was designed to include one employee per organization, the analytic dataset contained no intended repeated employee observations within organizations. The structural analyses were therefore conducted at the individual level without organization-clustered standard errors or a multilevel specification. The one-respondent-per-organization statement should be interpreted as a recruitment/screening rule rather than evidence of an organization-level construct.

For the measurement model, we examined indicator loadings, Cronbach’s *α*, rho_A, composite reliability (CR), average variance extracted (AVE), and HTMT for discriminant validity (Henseler et al., 2015). The structural assessment used VIF, standardized path coefficients, *R*^2^, and *f^2^*. Direct and indirect relationships were evaluated with 10,000 bootstrap resamples and two-tailed 95% bootstrap confidence intervals.

Specific indirect relationships were estimated for Digital Autonomy and Technostrain. A two-stage latent-variable interaction approach was used for the DWF × AAE terms in the Digital Autonomy and Technostrain equations. DWF and AAE main effects remained in each equation when the interaction term was added. Significant interactions were examined with simple slopes, and conditional indirect relationships were bootstrapped at different AAE levels.

We also examined potential common method variance because all focal variables came from the same respondents (Podsakoff et al., 2003; Podsakoff et al., 2024). Harman’s single-factor test served only as an initial diagnostic. We then inspected the correlations between ATCB and the five focal constructs and re-estimated the relevant structural equations with the marker entered as an additional control (Miller and Simmering, 2023; Simmering et al., 2015).

## 4 Results

### 4.1 Preliminary analysis and sample characteristics

The final sample comprised 462 employees. Women accounted for 52.2% of respondents and men for 46.3%. Participants aged 31–40 formed the largest age group (40.5%), followed by those aged 21–30 (32.3%). Most respondents held a bachelor’s degree (57.4%), while 24.7% held a master’s degree. Nonmanagerial employees represented 63.4% of the sample, compared with 18.2% front-line supervisors and 14.9% middle managers. In terms of work mode, 43.1% reported hybrid work, 36.4% mainly on-site work, and 20.6% mainly remote work. Full sample characteristics are reported in Supplementary Table S1.

No missing values remained on the focal items after screening. Skewness ranged from −0.47 to −0.06 and kurtosis from −0.86 to −0.46. Table 1 reports the descriptive statistics and zero-order correlations. Digital Work Flexibility was positively correlated with Digital Autonomy (*r* = 0.408, *p* < 0.001) and Technostrain (*r* = 0.316, *p* < 0.001). Digital Autonomy correlated positively with Employee Well-Being (*r* = 0.295, *p* < 0.001), while Technostrain correlated negatively with it (*r* = −0.430, *p* < 0.001). AAE correlated positively with Technostrain (*r* = 0.293, *p* < 0.001) and negatively with Employee Well-Being (*r* = −0.232, *p* < 0.001). The DWF-EWB correlation was small and nonsignificant (*r* = −0.044, *p* = 0.350). These are descriptive associations and do not test the proposed indirect or conditional relationships.

**Table 1:** Descriptive statistics and correlations.

Construct	Mean	SD	1	2	3	4	5
Digital work flexibility	4.71	1.62	—				
Digital autonomy	4.63	1.43	0.408^***^	—			
Technostrain	4.21	1.30	0.316^***^	0.091	—		
After-hours availability expectations	4.28	1.56	0.053	−0.088	0.293^***^	—	
Employee wellbeing	4.81	1.27	−0.044	0.295^***^	−0.430^***^	−0.232^***^	—

*N* = 462. Values below the diagonal are Pearson correlations. ^***^p < 0.001.

The common-method checks were treated as diagnostics rather than as proof that method variance was absent. In the unrotated single-factor analysis of the 46 focal items, the first factor accounted for 30.44% of the total variance. ATCB had acceptable internal consistency (Cronbach’s *α* = 0.818) and weak correlations with the five focal constructs, with absolute values from 0.004 to 0.054. With the marker included in the structural equations, the focal coefficients changed only trivially: DWF → Digital Autonomy changed from *β* = 0.400 to 0.399; DWF × AAE → Digital Autonomy from *β* = −0.123 to −0.122; DWF → Technostrain from *β* = 0.316 to 0.318; and DWF × AAE → Technostrain from *β* = 0.131 to 0.130. The Digital Autonomy → EWB and Technostrain → EWB estimates changed from *β* = 0.346 and *β* = −0.425 to 0.346 and −0.424. The marker was not significantly associated with the three main endogenous constructs. These results reduce, but do not eliminate, concern that the structural associations mainly reflect common measurement method (see Supplementary Table S9).

### 4.2 Measurement model assessment

We first assessed the reflective first-order measures and then evaluated Digital Autonomy, Technostrain, and Employee Well-Being as higher-order constructs with a two-stage procedure (Sarstedt et al., 2019). The assessment covered indicator reliability, internal consistency, convergent validity, and discriminant validity. As general PLS-SEM guidelines, we used loadings around 0.70 or above, reliability values above 0.70, and AVE above 0.50 as reference points.

All 46 focal-item loadings were between 0.787 and 0.930, and no item was removed on statistical grounds. Across the first-order constructs, Cronbach’s α ranged from 0.844 to 0.912, rho_A from 0.844 to 0.912, composite reliability from 0.907 to 0.932, and AVE from 0.665 to 0.865. These values indicate adequate internal consistency and convergent validity for the first-order measures (Table 2).

**Table 2:** First-order measurement model.

Construct	Items	Outer loading range	Cronbach’s α	rho_A	CR (rho_c)	AVE
Digital work flexibility	2	0.930–0.930	0.844	0.844	0.928	0.865
Decision-making autonomy	3	0.883–0.888	0.861	0.862	0.916	0.784
Work methods autonomy	3	0.878–0.897	0.866	0.866	0.918	0.788
Technostrain–anxiety	4	0.834–0.864	0.874	0.874	0.914	0.725
Technostrain–fatigue	4	0.866–0.876	0.894	0.894	0.926	0.758
Technostrain–scepticism	4	0.826–0.861	0.865	0.866	0.908	0.712
Technostrain–inefficacy	4	0.830–0.852	0.863	0.863	0.907	0.709
After-hours availability expectations	4	0.858–0.889	0.901	0.901	0.931	0.771
Life wellbeing	6	0.812–0.840	0.909	0.909	0.929	0.687
Workplace wellbeing	6	0.808–0.852	0.912	0.912	0.932	0.694
Psychological wellbeing	6	0.787–0.840	0.899	0.899	0.922	0.665

CR, composite reliability; AVE, average variance extracted.

HTMT was used to examine discriminant validity (Henseler et al., 2015). First-order values ranged from 0.038 to 0.848. None exceeded 0.85. The largest values involved dimensions belonging to the same higher-order construct: Decision-Making Autonomy with Work Methods Autonomy (0.848), Workplace Well-Being with Psychological Well-Being (0.832), and Technostrain Anxiety with Fatigue (0.824). These relatively high within-construct associations were expected and remained below the stricter 0.85 benchmark.

At the second stage, the lower-order construct scores served as indicators of the three reflective-reflective higher-order constructs (Sarstedt et al., 2019). Both Digital Autonomy dimensions loaded at 0.931. The four Technostrain dimensions ranged from 0.830 to 0.891, and the three Employee Well-Being dimensions from 0.890 to 0.913. Reliability exceeded 0.80 and AVE exceeded 0.75 for all three higher-order constructs. Supplementary Table S2 reports the full assessment.

Discriminant validity was also examined at the final construct level. Every HTMT value in Table 3 was below 0.85. The DWF-Digital Autonomy value was 0.483, the Technostrain-EWB value 0.484, and the AAE-Technostrain value 0.327. The modest DWF-Digital Autonomy HTMT is consistent with the intended distinction between when/where flexibility and decision/method autonomy.

**Table 3:** HTMT ratios at the final construct level.

Construct	1	2	3	4	5
Digital work flexibility	—				
Digital autonomy	0.483	—			
Technostrain	0.364	0.105	—		
After-hours availability expectations	0.060	0.100	0.327	—	
Employee wellbeing	0.050	0.340	0.484	0.259	—

Overall, the first- and higher-order measures met the stated reliability, convergent-validity, and discriminant-validity criteria, so the structural relationships were examined next.

### 4.3 Structural model and direct effects

Inner VIF values ranged from 1.005 to 1.325, indicating no material multicollinearity among the structural predictors (see Table 4).

**Table 4:** Direct effects and hypothesis testing.

Hypothesis	Structural path	*β*	Bootstrap SE	*t*	95% bootstrap CI	*f^2^*	Result
H1a	DWF → Digital autonomy	0.400	0.042	9.548	[0.317, 0.482]	0.195	Supported
H1b	DWF → Technostrain	0.316	0.042	7.466	[0.233, 0.400]	0.122	Supported
H2a	Digital autonomy → EWB	0.346	0.042	8.225	[0.265, 0.428]	0.141	Supported
H2b	Technostrain → EWB	−0.425	0.041	10.289	[−0.506, −0.342]	0.214	Supported
—	DWF → EWB	−0.047	0.045	1.044	[−0.134, 0.041]	0.002	n.s.

*N* = 462. Bootstrap = 10,000 resamples; two-tailed 95% bootstrap confidence intervals. The DWF → EWB path was estimated to avoid imposing full mediation but was not associated with a directional hypothesis.

The model accounted for 19.3% of the variance in Digital Autonomy (*R*^2^ = 0.193), 19.3% in Technostrain (*R*^2^ = 0.193), and 30.4% in Employee Well-Being (*R*^2^ = 0.304). The two first-stage equations included DWF, AAE, and their interaction; the EWB equation included DWF, Digital Autonomy, Technostrain, and AAE. Supplementary Table S3 reports *R*^2^ and adjusted *R*^2^ values.

AAE was retained as a non-hypothesized predictor of Employee Well-Being. Its direct association with EWB was negative but nonsignificant (*β* = −0.074, *p* = 0.071). No separate hypothesis was proposed for this path because the focal role of AAE in the study is conditional rather than its average association with well-being.

With 10,000 bootstrap resamples, Digital Work Flexibility was positively associated with Digital Autonomy (*β* = 0.400, *t* = 9.548, *p* < 0.001, 95% bootstrap CI [0.317, 0.482]), supporting H1a. The corresponding *f^2^* was 0.195.

Digital Work Flexibility was also positively associated with Technostrain (*β* = 0.316, *t* = 7.466, *p* < 0.001, 95% bootstrap CI [0.233, 0.400]), supporting H1b (*f^2^* = 0.122). Thus, higher reported flexibility coincided with both higher Digital Autonomy and higher Technostrain.

Digital Autonomy was positively associated with Employee Well-Being (*β* = 0.346, *t* = 8.225, *p* < 0.001, 95% bootstrap CI [0.265, 0.428]), supporting H2a (*f^2^* = 0.141). Technostrain was negatively associated with Employee Well-Being (*β* = −0.425, *t* = 10.289, *p* < 0.001, 95% bootstrap CI [−0.506, −0.342]), supporting H2b (*f^2^* = 0.214).

Once Digital Autonomy, Technostrain, and AAE were included, the direct DWF-EWB association was small and nonsignificant (*β* = −0.047, *t* = 1.044, *p* = 0.296, 95% bootstrap CI [−0.134, 0.041], *f^2^* = 0.002). This path was estimated without a directional hypothesis; its nonsignificance is considered alongside the two specific indirect relationships reported below.

### 4.4 Specific indirect relationships

The two specific indirect relationships were estimated with 10,000 bootstrap resamples. Because AAE is part of the first-stage interaction model, the estimates in this section refer to the mean level of AAE.

The indirect relationship through Digital Autonomy was positive and significant (*β* = 0.139, bootstrap SE = 0.023, 95% bootstrap CI [0.096, 0.187]), supporting H3a. At mean AAE, higher DWF was associated with higher Digital Autonomy, which was in turn associated with higher Employee Well-Being.

The indirect relationship through Technostrain was negative and significant (*β* = −0.135, bootstrap SE = 0.022, 95% bootstrap CI [−0.180, −0.092]), supporting H3b. At mean AAE, higher DWF was also associated with higher Technostrain, which was associated with lower Employee Well-Being.

The two indirect estimates were similar in magnitude but opposite in sign. Their summed net indirect effect was *β* = 0.004 (95% bootstrap CI [−0.058, 0.068]) and was not significant. This net quantity was an additional, non-hypothesized analysis; H3a and H3b are evaluated from the two specific indirect relationships. Section 4.6 reports the conditional estimates across AAE levels.

### 4.5 Moderating relationships

Moderation was examined by estimating a DWF × AAE term in each first-stage equation. Predictor scores were standardized before forming the interaction, and inference used 10,000 bootstrap resamples.

The DWF and AAE main effects were retained in both equations. AAE was negatively associated with Digital Autonomy (*β* = −0.104, *p* = 0.014) and positively associated with Technostrain (*β* = 0.270, *p* < 0.001). These non-hypothesized estimates describe the average associations of AAE with the two first-stage outcomes. The hypotheses concern whether the DWF associations differ across AAE levels, so the interaction terms are the focal tests (see Table 5).

**Table 5:** Indirect and moderating relationships.

Panel A Specific indirect relationships						
Hypothesis	Indirect path	*β*	Bootstrap SE	*t*	95% bootstrap CI	Result
H3a	DWF → Digital autonomy → EWB	0.139	0.023	5.984	[0.096, 0.187]	Supported
H3b	DWF → Technostrain → EWB	−0.135	0.022	6.022	[−0.180, −0.092]	Supported
—	Net indirect effect	0.004	0.032	0.126	[−0.058, 0.068]	n.s.
*N* = 462. Bootstrap = 10,000 resamples. Two-tailed 95% bootstrap confidence intervals are reported. Because the first-stage paths are moderated by AAE, the specific indirect effects shown here are evaluated at the mean level of AAE. The net indirect effect was examined as an additional analysis and was not associated with a separate hypothesis.						

Panel B Moderating relationships involving after-hours availability expectations								
Hypothesis	Interaction path	*β*	Bootstrap SE	*t*	*p*	95% bootstrap CI	*f^2^*	Result
H4a	DWF × AAE → Digital autonomy	−0.123	0.043	2.84	0.005	[−0.200, −0.041]	0.018	Supported
H4b	DWF × AAE → Technostrain	0.131	0.043	3.03	0.003	[0.046, 0.215]	0.020	Supported

*N* = 462. Bootstrap = 10,000 resamples. Predictor scores were standardized before the interaction term was constructed. Two-tailed 95% bootstrap confidence intervals are reported.

For Digital Autonomy, the DWF × AAE coefficient was negative (*β* = −0.123, bootstrap SE = 0.043, *t* = 2.84, *p* = 0.005, 95% bootstrap CI [−0.200, −0.041]), supporting H4a. The incremental effect size was *f^2^* = 0.018.

The simple slopes show the conditional pattern. The positive DWF-Digital Autonomy association was *β* = 0.523 at low AAE (−1 SD; 95% bootstrap CI [0.406, 0.634]), *β* = 0.400 at the mean (95% bootstrap CI [0.318, 0.482]), and *β* = 0.277 at high AAE (+1 SD; 95% bootstrap CI [0.163, 0.396]). Figure 2 displays the same pattern: the positive association was smaller at higher AAE.

**Figure 2:**
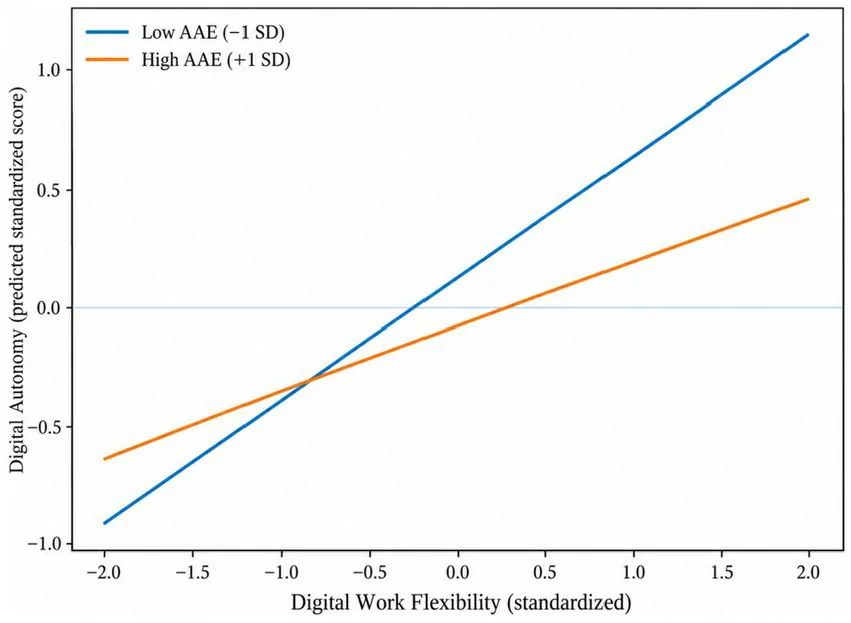
Simple-slope pattern for digital work flexibility and digital autonomy at Low and high after-hours availability expectations.

For Technostrain, the interaction coefficient was positive (*β* = 0.131, bootstrap SE = 0.043, *t* = 3.03, *p* = 0.003, 95% bootstrap CI [0.046, 0.215]), supporting H4b. Its incremental *f^2^* was 0.020.

Using moderation-specific reference values, *f^2^* = 0.018 and 0.020 fall above the medium benchmark and below the large benchmark reported for interaction effects in PLS-SEM (Hair et al., 2022).

The DWF-Technostrain association was *β* = 0.185 at low AAE (95% bootstrap CI [0.068, 0.301]), *β* = 0.316 at the mean (95% bootstrap CI [0.233, 0.398]), and *β* = 0.448 at high AAE (95% bootstrap CI [0.326, 0.565]). Figure 3 illustrates this ordering across AAE levels.

**Figure 3:**
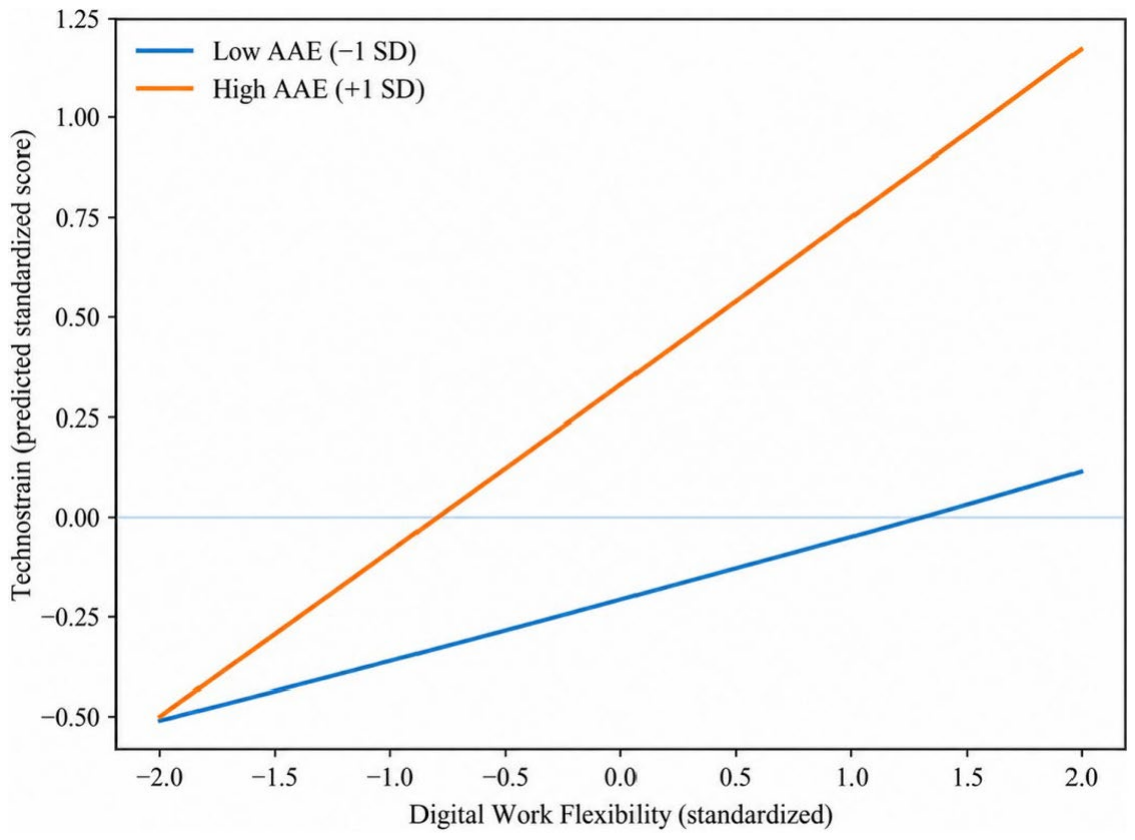
Simple-slope pattern for digital work flexibility–technostrain by AAE level.

The two interactions point in opposite directions. Across higher AAE levels, the positive DWF-Digital Autonomy association was smaller, whereas the positive DWF-Technostrain association was larger. Supplementary Table S4 reports the full simple-slope estimates; Section 4.6 turns to the corresponding conditional indirect relationships.

### 4.6 Conditional indirect relationships and conditional Total associations

Conditional indirect relationships were estimated at low (−1 SD), mean, and high (+1 SD) AAE using 10,000 bootstrap resamples. We also examined indices of moderated mediation for each pathway (see Table 6).

**Table 6:** Conditional indirect relationships and indices of moderated mediation.

Panel A Conditional Indirect Relationships at Different Levels of After-hours Availability Expectations				
Indirect pathway	AAE level	Indirect effect (*β*)	Bootstrap SE	95% Bootstrap CI
DWF → Digital autonomy → EWB	Low (−1 SD)	0.181	0.031	[0.124, 0.245]
	Mean	0.139	0.023	[0.096, 0.187]
	High (+1 SD)	0.096	0.024	[0.052, 0.147]
DWF → Technostrain → EWB	Low (−1 SD)	−0.079	0.026	[−0.131, −0.029]
	Mean	−0.135	0.022	[−0.180, −0.092]
	High (+1 SD)	−0.190	0.034	[−0.260, −0.129]
Net indirect effect	Low (−1 SD)	0.102	0.039	[0.029, 0.181]
	Mean	0.004	0.032	[−0.058, 0.068]
	High (+1 SD)	−0.094	0.042	[−0.178, −0.012]

Panel B Indices of moderated mediation					
Hypothesis	Conditional indirect pathway	Index	Bootstrap SE	95% Bootstrap CI	Result
H5a	DWF → Digital autonomy → EWB	−0.043	0.015	[−0.074, −0.014]	Supported
H5b	DWF → Technostrain → EWB	−0.056	0.020	[−0.098, −0.018]	Supported
—	Net conditional indirect effect	−0.098	0.025	[−0.149, −0.051]	Additional analysis

For Digital Autonomy, the positive indirect relationship was *β* = 0.181 at low AAE (bootstrap SE = 0.031, 95% bootstrap CI [0.124, 0.245]), *β* = 0.139 at the mean (95% bootstrap CI [0.096, 0.187]), and *β* = 0.096 at high AAE (95% bootstrap CI [0.052, 0.147]). The index of moderated mediation was negative and significant (index = −0.043, bootstrap SE = 0.015, 95% bootstrap CI [−0.074, −0.014]). The positive indirect association was smaller at higher AAE, supporting H5a.

The Technostrain pathway showed the reverse ordering. The negative indirect relationship was *β* = −0.079 at low AAE (bootstrap SE = 0.026, 95% bootstrap CI [−0.131, −0.029]), *β* = −0.135 at the mean (95% bootstrap CI [−0.180, −0.092]), and *β* = −0.190 at high AAE (95% bootstrap CI [−0.260, −0.129]). Its index of moderated mediation was also significant (index = −0.056, bootstrap SE = 0.020, 95% bootstrap CI [−0.098, −0.018]). The negative indirect association was larger in magnitude at higher AAE, supporting H5b.

For an additional descriptive comparison, the two conditional indirect relationships were summed. The net indirect association was positive at low AAE (*β* = 0.102, 95% bootstrap CI [0.029, 0.181]), near zero at the mean (*β* = 0.004, 95% bootstrap CI [−0.058, 0.068]), and negative at high AAE (*β* = −0.094, 95% bootstrap CI [−0.178, −0.012]). The index for this difference across AAE levels was significant (index = −0.098, 95% bootstrap CI [−0.149, −0.051]). These estimates describe a change in the sign of the net indirect association only; they do not, by themselves, establish a reversal in the overall DWF-EWB association.

To distinguish the conditional net indirect association from the overall DWF–EWB association, conditional total associations were bootstrapped from the specified model, in which the direct DWF–EWB path was not moderated by AAE. The conditional total association was *β* = 0.055 at low AAE (95% bootstrap CI [−0.047, 0.160]), *β* = −0.043 at mean AAE (95% bootstrap CI [−0.135, 0.051]), and *β* = −0.141 at high AAE (95% bootstrap CI [−0.242, −0.035]). Thus, the low- and mean-AAE total associations were not statistically distinguishable from zero, whereas the high-AAE total association was negative. These results do not support describing the pattern as a simple positive-to-negative reversal of the overall relationship. Supplementary Table S5 reports the conditional total-association analysis.

### 4.7 Sensitivity and robustness checks

Because the original theoretical model did not hypothesize moderation of the direct DWF–EWB path, the specified model treated that direct path as unmoderated. To answer the reviewer’s question directly, we estimated an exploratory expanded model that added DWF × AAE → EWB. The interaction was negative (*β* = −0.082, 95% bootstrap CI [−0.160, −0.000]). Because this path was not part of the *a priori* hypotheses, it is interpreted only as a sensitivity analysis rather than as confirmatory evidence.

When the conditional total associations were recomputed in the expanded model, the total association was *β* = 0.126 at low AAE (95% bootstrap CI [−0.000, 0.246]), *β* = −0.051 at mean AAE (95% bootstrap CI [−0.144, 0.044]), and *β* = −0.228 at high AAE (95% bootstrap CI [−0.363, −0.084]). The same substantive conclusion follows: the low-AAE estimate was not clearly distinguishable from zero, while the high-AAE association was negative. Supplementary Table S5 reports the specified-model totals, the exploratory direct-path moderation, and the expanded-model totals.

As a robustness check, the focal equations were re-estimated using standardized unit-weighted composite scores and OLS. Because the indicators showed relatively consistent loadings, the standardized unit-weighted composites were highly correlated with the PLS-based construct scores. Therefore, similar structural estimates were expected when the model was re-estimated using the alternative scoring approach. Most path estimates were unchanged to three decimal places, and the conditional indirect pattern was reproduced (Supplementary Table S6).

The model was also re-estimated with the Attitude Toward the Color Blue marker included as a control for potential method-related variance. The focal path coefficients remained close to those obtained from the main model (Supplementary Table S9). Although this comparison does not eliminate the possibility of common method variance, the marker adjustment produced little change in the substantive estimates.

The structural model was also re-estimated with gender, age, organizational tenure, managerial position, and weekly working hours as controls. The focal coefficients remained close to the main-model estimates: DWF-Digital Autonomy *β* = 0.409 (*p* < 0.001), DWF-Technostrain *β* = 0.307 (*p* < 0.001), Digital Autonomy-EWB *β* = 0.355 (*p* < 0.001), and Technostrain-EWB *β* = −0.427 (*p* < 0.001). The interaction coefficients were −0.121 for Digital Autonomy (*p* = 0.006) and 0.131 for Technostrain (*p* = 0.003). The direct DWF-EWB association remained nonsignificant (*β* = −0.057, *p* = 0.217). Supplementary Table S7 gives the full comparison.

Finally, the EWB equation was estimated separately for Life, Workplace, and Psychological Well-Being. Digital Autonomy was positively associated with all three dimensions (*β* = 0.264, 0.340, and 0.334; all *p* < 0.001), while Technostrain was negatively associated with all three (*β* = −0.365, −0.413, and −0.373; all *p* < 0.001). The direct DWF association was nonsignificant in each case. The consistency of this pattern supports the use of higher-order Employee Well-Being as the primary outcome while showing that the result is not concentrated in one domain (Supplementary Table S8).

## 5 Discussion

### 5.1 Interpretation of the findings

The results are difficult to summarize with a single coefficient. Digital Work Flexibility was positively associated with both Digital Autonomy and Technostrain, and those two variables were associated with Employee Well-Being in opposite directions. At mean AAE, the positive and negative specific indirect relationships were similar in magnitude and nearly offset one another. Accordingly, the small direct DWF-EWB association should not be interpreted as evidence that flexibility was unrelated to employee experience. The data instead show two statistically meaningful indirect associations with opposite signs. Because the study is cross-sectional, these estimates are interpreted as associations consistent with the proposed model rather than as evidence of a confirmed temporal mediation process.

The pattern was also consistent across the three domains of Employee Well-Being. Digital Autonomy was positively associated with Life, Workplace, and Psychological Well-Being, whereas Technostrain was negatively associated with each domain. Similar associations have been reported for autonomy in digitally mediated work (Wan et al., 2024; Olivo et al., 2025) and for technostress in relation to well-being and work-life outcomes (Bottaro et al., 2024; Mansuroğlu and Smith, 2026). The consistency across the three domains suggests that the opposing pathways were not driven by a single component of Employee Well-Being.

Digital Work Flexibility should therefore not be interpreted only through its possible association with employee control. Employees reporting greater flexibility over when and where work was performed also reported greater discretion over work decisions and methods, while simultaneously reporting higher Technostrain. The study does not establish that flexibility caused either experience, but the coexistence of these associations helps explain why a weak overall DWF-EWB relationship can mask opposing patterns within the same cross-sectional model (Shifrin and Michel, 2022; Harrop et al., 2026).

Perceived AAE helps describe when these different associations are more or less prominent. At lower AAE, the autonomy-related association was relatively stronger; at higher AAE, the technostrain-related association was relatively stronger. In the specified model, the conditional total association was not statistically distinguishable from zero at low or mean AAE but was negative at high AAE. This conditional pattern is consistent with the Autonomy Paradox in the limited sense that technology-enabled flexibility can coexist with perceived expectations of continued availability (Mazmanian et al., 2013). It should not be read as evidence of a symmetric overall relationship reversal or as evidence that AAE directly changes employees’ psychology, because temporal ordering and actual after-hours connectivity behavior were not observed.

### 5.2 Theoretical implications

Prior research has separately established that digitally enabled and flexible work can be associated with both resources and strain, while autonomy-paradox research has shown that greater discretion can coexist with expectations of continual connectivity (Mazmanian et al., 2013; Shifrin and Michel, 2022; Kossek et al., 2023). The contribution of the present study is to bring these ideas into a more specific conditional model. Relative to flexible-work studies that examine outcomes at a broader level and autonomy-paradox studies centered on continual accessibility, we distinguish temporal/spatial flexibility from decision/method autonomy, estimate the autonomy-related and technostrain-related indirect associations simultaneously, and examine whether employees’ perceived AAE shifts the strength of the two first-stage associations in opposite directions. This positioning does not claim to resolve the broader flexible-work literature; it identifies a more precise conditional pattern that can be examined with longitudinal and behavioral designs.

A related distinction concerns the level of analysis. Firm-level research on Chinese listed companies has examined digital transformation in relation to corporate performance and the strategic role of coopetition (Wang and Lim, 2025). The present study addresses a different analytical level: rather than organizational performance mechanisms, it examines employee-level autonomy-related and strain-related experiences associated with digitally enabled flexibility. This cross-level contrast matters because organizational gains associated with digital transformation cannot be assumed to translate uniformly into favorable employee experiences.

The role of perceived AAE further sharpens this contribution. AAE did not represent an organization-level norm or actual communication behavior; it represented employees’ perceptions of supervisor expectations. Within that scope, the DWF-Digital Autonomy association was weaker and the DWF-Technostrain association stronger at higher AAE. This is consistent with the idea that formal flexibility and perceived accessibility pressure can coexist, while avoiding the stronger claim that the present cross-sectional data demonstrate how employees psychologically reinterpret flexibility over time (Mazmanian et al., 2013).

Another implication concerns construct boundaries. Digital Work Flexibility concerns when and where work is performed, whereas Digital Autonomy in this study concerns discretion over decisions and methods. The Digital Autonomy measure therefore does not operationalize the full autonomy paradox, scheduling autonomy, or boundary control. Maintaining this distinction reduces conceptual overlap between DWF and Digital Autonomy and makes the empirical contribution more precise: flexibility over time and location was related, but not equivalent, to decision and method discretion.

### 5.3 Practical implications

For organizations, the findings suggest that the practical meaning of Digital Work Flexibility may depend on the expectations surrounding its use. Greater freedom over when and where work is performed can coexist with perceived pressure to remain reachable after normal working hours. Clearer communication about urgency, expected response windows, and the legitimacy of disconnection may therefore represent promising managerial practices. However, the present study did not test communication-boundary policies or supervisor training as interventions, so these suggestions should be treated as implications for practice that require direct evaluation in intervention research (Piszczek, 2017; He et al., 2024).

As remote, hybrid, and digitally enabled work arrangements expand, formal flexibility policies alone may not capture employees’ day-to-day experience. Perceived expectations regarding response speed, after-hours messages, and unwritten availability requirements may accompany the same formal flexibility arrangement. Organizations may therefore consider clearer communication boundaries, while recognizing that the effectiveness of such practices cannot be inferred from this cross-sectional study and should be tested prospectively (Nilsen et al., 2026).

### 5.4 Limitations and future research

Several features of the research design require caution. All focal constructs were reported by the same employees at a single time point. The data therefore do not establish the temporal ordering required for a causal mediation claim, and reverse relationships remain plausible; for example, employees with lower well-being may perceive greater Technostrain, less autonomy, or their flexibility differently. Cross-sectional indirect estimates can differ substantially from longitudinal mediation processes (Maxwell et al., 2011). Anonymous responding and the marker-variable procedure reduce, but do not eliminate, concerns about common method variance (Podsakoff et al., 2003; Podsakoff et al., 2024). Future research should use longitudinal, cross-lagged, experience-sampling, or multi-source designs before drawing stronger conclusions about the proposed psychological sequence.

The measurement of AAE raises related issues of level, perspective, and mechanism. AAE reflects employees’ perceptions of supervisor expectations (Derks et al., 2015), whereas supervisors’ actual communication behavior, after-hours message volume, response speed, nonwork-time technology use, digital workload, task interdependence, and formal organizational connectivity policies were not measured. AAE should therefore not be treated as equivalent to actual connectivity, and robustness analyses involving these unmeasured behavioral or job-design variables could not be conducted with the present survey. In addition, one employee was retained from each organization, which prevents estimation of shared team-, supervisor-, or organization-level availability norms. Research using supervisor-employee dyads, digital trace indicators, experience sampling, or multiple employees within the same team could distinguish individual perceptions from actual behavior and shared availability climate (Piszczek, 2017; He et al., 2024; Nilsen et al., 2026).

Technostrain and Employee Well-Being were examined as higher-order constructs. This was useful for testing the broader resource–strain pattern, but aggregation may conceal differences among their dimensions. Anxiety, Fatigue, Scepticism, and Inefficacy need not have identical relationships with Employee Well-Being, just as Life, Workplace, and Psychological Well-Being may differ in the magnitude or timing of their associations with Digital Work Flexibility and Digital Autonomy. The dimension-level analyses in this study showed similar overall directions across the three well-being domains, but future studies could examine the individual dimensions more directly and identify whether particular forms of digital work are associated more strongly with specific forms of Technostrain or well-being (Kotek and Vranjes, 2025; Mansuroğlu and Smith, 2026).

The measures of Digital Work Flexibility and Digital Autonomy also impose clear scope boundaries. DWF captures a deliberately narrow combination of perceived temporal and spatial flexibility (Jones et al., 2008), whereas Digital Autonomy captures Decision-Making Autonomy and Work Methods Autonomy (Morgeson and Humphrey, 2006). Work Scheduling Autonomy was omitted to avoid overlap with the temporal component of DWF. Although the two-item DWF measure retained both items from the selected operationalization and showed adequate measurement properties in this sample, two items provide less construct breadth than a multidimensional flexibility measure. Future work could examine temporal and spatial flexibility separately, include additional autonomy facets, and test boundary-management constructs directly. Generalizability is also limited because detailed industry and occupational-function categories were not retained; the findings should therefore be applied cautiously beyond the Jiangxi full-time employees reached through the regional SME-related recruitment network.

## 6 Conclusion

Digital Work Flexibility cannot be understood from flexibility alone. In this cross-sectional sample, employees who reported greater flexibility also reported greater Digital Autonomy and greater Technostrain, and those two experiences showed opposite associations with Employee Well-Being. These findings identify a coexistence of statistically meaningful resource-related and strain-related patterns rather than a single uniformly positive or negative relationship.

Perceived AAE further distinguished these patterns: the autonomy-related association was weaker and the technostrain-related association stronger at higher AAE. The result is best interpreted as a conditional pattern of associations, not as evidence that the overall DWF-EWB relationship necessarily reverses or that a causal mechanism has been established. Longitudinal and behavioral evidence is needed to determine whether and how these relationships unfold over time.

## Data Availability

The raw data supporting the conclusions of this article will be made available by the authors, without undue reservation.

## References

[ref1] Allen T. D. Golden T. D. Shockley K. M. (2015). How effective is telecommuting? Assessing the status of our scientific findings. Psychol. Sci. Public Interest 16, 40–68. doi: 10.1177/1529100615593273, 26403188

[ref2] Allen T. D. Johnson R. C. Kiburz K. M. Shockley K. M. (2013). Work–family conflict and flexible work arrangements: deconstructing flexibility. Pers. Psychol. 66, 345–376. doi: 10.1111/peps.12012

[ref3] Allen T. D. Merlo K. Lawrence R. C. Slutsky J. Gray C. E. (2021). Boundary management and work-nonwork balance while working from home. Appl. Psychol. 70, 60–84. doi: 10.1111/apps.12300

[ref4] Bakker A. B. Demerouti E. (2017). Job demands–resources theory: taking stock and looking forward. J. Occup. Health Psychol. 22, 273–285. doi: 10.1037/ocp0000056 27732008

[ref5] Bakker A. B. Demerouti E. (2024). Job demands-resources theory: frequently asked questions. J. Occup. Health Psychol. 29, 188–200. doi: 10.1037/ocp0000376, 38913705

[ref6] Bakker A. B. Demerouti E. Sanz-Vergel A. (2023). Job demands–resources theory: ten years later. Annu. Rev. Organ. Psychol. Organ. Behav. 10, 25–53. doi: 10.1146/annurev-orgpsych-120920-053933

[ref7] Barber L. K. Conlin A. L. Santuzzi A. M. (2019). Workplace telepressure and work–life balance outcomes: the role of work recovery experiences. Stress. Health 35, 350–362. doi: 10.1002/smi.2864, 30882979

[ref8] Barber L. K. Santuzzi A. M. (2015). Please respond ASAP: workplace telepressure and employee recovery. J. Occup. Health Psychol. 20, 172–189. doi: 10.1037/a0038278 25365629

[ref9] Baum L. Rau R. (2024). Balancing work and private life: when does workplace flexibility really help? New insights into the interaction effect of working from home and job autonomy. Front. Organ. Psychol. 2:1271726. doi: 10.3389/forgp.2024.1271726

[ref10] Becker J. Lanzl J. (2023). Segmentation preference and technostress: integrators’ vs segmenters’ experience of technology-induced demands and related spill-over effects. Inf. Manag. 60:103811. doi: 10.1016/j.im.2023.103811

[ref11] Bottaro R. De Giovanni K. Faraci P. (2024). The extent to which technostress is related to employees’ work-life fit: a multilevel meta-analysis. Workplace Health & Safety 72, 421–430. doi: 10.1177/21650799241264317, 39126189

[ref12] Brislin R. W. (1970). Back-translation for cross-cultural research. J. Cross-Cult. Psychol. 1, 185–216. doi: 10.1177/135910457000100301

[ref13] Charalampous M. Grant C. A. Tramontano C. Michailidis E. (2019). Systematically reviewing remote e-workers’ well-being at work: a multidimensional approach. Eur. J. Work Organ. Psychol. 28, 51–73. doi: 10.1080/1359432X.2018.1541886

[ref14] Demerouti E. (2025). Job demands-resources and conservation of resources theories: how do they help to explain employee well-being and future job design? J. Bus. Res. 192:115296. doi: 10.1016/j.jbusres.2025.115296

[ref15] Derks D. van Duin D. Tims M. Bakker A. B. (2015). Smartphone use and work–home interference: the moderating role of social norms and employee work engagement. J. Occup. Organ. Psychol. 88, 155–177. doi: 10.1111/joop.12083

[ref16] Faul F. Erdfelder E. Buchner A. Lang A.-G. (2009). Statistical power analyses using G*power 3.1: tests for correlation and regression analyses. Behav. Res. Methods 41, 1149–1160. doi: 10.3758/BRM.41.4.1149, 19897823

[ref17] Hair J. F. Hult G. T. M. Ringle C. M. Sarstedt M. (2022). A primer on Partial least Squares Structural Equation Modeling (PLS-SEM). 3rd Edn. Thousand Oaks, CA, USA: SAGE.

[ref18] Hair J. F. Risher J. J. Sarstedt M. Ringle C. M. (2019). When to use and how to report the results of PLS-SEM. Eur. Bus. Rev. 31, 2–24. doi: 10.1108/EBR-11-2018-0203

[ref19] Harrop N. Jiang L. Overall N. (2026). A meta-analysis of antecedents and outcomes of flexible working arrangements. J. Organ. Behav. 47, 208–236. doi: 10.1002/job.2896

[ref20] He X. Gao Q. Cao Y. Bian R. Wang X.-H. F. (2024). ‘Always online’: how and when task interdependence and dispositional workplace anxiety affect workplace telepressure after hours. PsyCh Journal 13, 639–653. doi: 10.1002/pchj.747, 38530885 PMC11317195

[ref21] Henseler J. Ringle C. M. Sarstedt M. (2015). A new criterion for assessing discriminant validity in variance-based structural equation modeling. J. Acad. Mark. Sci. 43, 115–135. doi: 10.1007/s11747-014-0403-8

[ref22] Jones B. L. Scoville D. P. Hill E. J. Childs G. Leishman J. M. Nally K. S. (2008). Perceived versus used workplace flexibility in Singapore: predicting work–family fit. J. Fam. Psychol. 22, 774–783. doi: 10.1037/a0013181, 18855513

[ref23] Kossek E. E. Perrigino M. B. Lautsch B. A. (2023). Work-life flexibility policies from a boundary control and implementation perspective: a review and research framework. J. Manag. 49, 2062–2108. doi: 10.1177/01492063221140354

[ref24] Kotek M. Vranjes I. (2025). When do employees perceive technology as stressful? A meta-analysis of work-related technostress antecedents. Inf. Manag. 62:104203. doi: 10.1016/j.im.2025.104203

[ref25] Kumar P. S. (2024). Technostress: a comprehensive literature review on dimensions, impacts, and management strategies. Comput. Hum. Behav. Rep. 16:100475. doi: 10.1016/j.chbr.2024.100475

[ref26] Mansuroğlu E. Smith A. P. (2026). Technostress and employee well-being: a systematic review of empirical evidence. Comput. Hum. Behav. Rep. 21:100941. doi: 10.1016/j.chbr.2026.100941

[ref27] Marsh E. Perez Vallejos E. Spence A. (2024). Digital workplace technology intensity: qualitative insights on employee wellbeing impacts of digital workplace job demands. Front. Organ. Psychol. 2:1392997. doi: 10.3389/forgp.2024.1392997

[ref28] Maxwell S. E. Cole D. A. Mitchell M. A. (2011). Bias in cross-sectional analyses of longitudinal mediation: partial and complete mediation under an autoregressive model. Multivar. Behav. Res. 46, 816–841. doi: 10.1080/00273171.2011.606716, 26736047

[ref29] Mazmanian M. Orlikowski W. J. Yates J. (2013). The autonomy paradox: the implications of mobile email devices for knowledge professionals. Organ. Sci. 24, 1337–1357. doi: 10.1287/orsc.1120.0806

[ref30] Mellner C. (2016). After-hours availability expectations, work-related smartphone use during leisure, and psychological detachment: the moderating role of boundary control. Int. J. Work. Health Manag. 9, 146–164. doi: 10.1108/IJWHM-07-2015-0050

[ref31] Miller B. K. Simmering M. J. (2023). Attitude toward the color blue: an ideal marker variable. Organ. Res. Methods 26, 409–440. doi: 10.1177/10944281221075361

[ref32] Morgeson F. P. Humphrey S. E. (2006). The work design questionnaire (WDQ): developing and validating a comprehensive measure for assessing job design and the nature of work. J. Appl. Psychol. 91, 1321–1339. doi: 10.1037/0021-9010.91.6.1321 17100487

[ref33] Nilsen W. Nordberg T. Lescoeur K. Ingelsrud M. H. Egeland C. (2026). What do we know about limiting after-hours availability expectations and work-related connectivity? A systematic review of interventions and policies. Scand. J. Work Environ. Health 52, 206–218. doi: 10.5271/sjweh.4277, 41648902 PMC13147545

[ref34] Olivo I. Ghezzi V. Marzocchi I. Fusco L. Spinella F. Isolani S. . (2025). Beyond boundaries: how autonomy and technology promote work engagement and well-being in remote workers. Front. Public Health 13:1659185. doi: 10.3389/fpubh.2025.1659185, 40988669 PMC12450924

[ref35] Parker S. K. Grote G. (2022). Automation, algorithms, and beyond: why work design matters more than ever in a digital world. Appl. Psychol. 71, 1171–1204. doi: 10.1111/apps.12241

[ref36] Piszczek M. M. (2017). Boundary control and controlled boundaries: organizational expectations for technology use at the work–family interface. J. Organ. Behav. 38, 592–611. doi: 10.1002/job.2153

[ref37] Podsakoff P. M. MacKenzie S. B. Lee J.-Y. Podsakoff N. P. (2003). Common method biases in behavioral research: a critical review of the literature and recommended remedies. J. Appl. Psychol. 88, 879–903. doi: 10.1037/0021-9010.88.5.879, 14516251

[ref38] Podsakoff P. M. Podsakoff N. P. Williams L. J. Huang C. Yang J. (2024). Common method bias: it’s bad, it’s complex, it’s widespread, and it’s not easy to fix. Annu. Rev. Organ. Psychol. Organ. Behav. 11, 17–61. doi: 10.1146/annurev-orgpsych-110721-040030

[ref39] Salanova M. Llorens S. Cifre E. (2013). The dark side of technologies: technostress among users of information and communication technologies. Int. J. Psychol. 48, 422–436. doi: 10.1080/00207594.2012.680460 22731610

[ref40] Sarstedt M. Hair J. F. Cheah J.-H. Becker J.-M. Ringle C. M. (2019). How to specify, estimate, and validate higher-order constructs in PLS-SEM. Australas. Mark. J. 27, 197–211. doi: 10.1016/j.ausmj.2019.05.003

[ref41] Scholze A. Hecker A. (2024). The job demands-resources model as a theoretical lens for the bright and dark side of digitization. Comput. Hum. Behav. 155:108177. doi: 10.1016/j.chb.2024.108177

[ref42] Shifrin N. V. Michel J. S. (2022). Flexible work arrangements and employee health: a meta-analytic review. Work Stress 36, 60–85. doi: 10.1080/02678373.2021.1936287

[ref43] Simmering M. J. Fuller C. M. Richardson H. A. Ocal Y. Atinc G. M. (2015). Marker variable choice, reporting, and interpretation in the detection of common method variance: a review and demonstration. Organ. Res. Methods 18, 473–511. doi: 10.1177/1094428114560023

[ref44] Simon A. C. Buzás B. B. Rosta-Filep O. Faragó K. Pachner O. C. Kiss O. E. (2024). The indirect impact of the technostress subfactors on the satisfaction and desire to work from home. Front. Psychol. 15:1417916. doi: 10.3389/fpsyg.2024.1417916, 38933584 PMC11203091

[ref45] Tarafdar M. Cooper C. L. Stich J.-F. (2019). The technostress trifecta—techno eustress, techno distress and design: theoretical directions and an agenda for research. Inf. Syst. J. 29, 6–42. doi: 10.1111/isj.12169

[ref46] Wan Z. Zhang L. Wang L. Zhang F. (2024). Navigating autonomy: Unraveling the dual influence of job autonomy on workplace well-being in the gig economy. Front. Psychol. 15:1369495. doi: 10.3389/fpsyg.2024.1369495, 39118837 PMC11307207

[ref47] Wang Y. Lee E. L. W. (2023). Realizing excellence in enterprise management through digital innovation. J. Logist. Inform. Serv. Sci. 10, 197–211. doi: 10.33168/JLISS.2023.0214

[ref48] Wang Y. Lim W. L. (2025). The interplay of digital transformation, coopetition strategy, and corporate performance: insights from Chinese listed companies. Financ. Res. Lett. 85:108043. doi: 10.1016/j.frl.2025.108043

[ref49] Wang B. Liu Y. Qian J. Parker S. K. (2021). Achieving effective remote working during the COVID-19 pandemic: a work design perspective. Appl. Psychol. 70, 16–59. doi: 10.1111/apps.12290, 33230359 PMC7675760

[ref50] Wang Y. Wu D. (2025). The power of duality: exploring the multi-dimensional impact of coopetition strategy on core competency in new start-up SMEs. Int. Rev. Financ. Anal. 108:104654. doi: 10.1016/j.irfa.2025.104654

[ref51] Zheng X. Zhu W. Zhao H. Zhang C. (2015). Employee well-being in organizations: theoretical model, scale development, and cross-cultural validation. J. Organ. Behav. 36, 621–644. doi: 10.1002/job.1990

