# Supplementary material for: When flexibility feels like control: competing autonomy and Technostrain pathways linking digital work flexibility to employee well-being
Source: Front Psychol. 2026 Oct 22;17:1977600. doi: 10.3389/fpsyg.2026.1977600 (PMC13735578; doi:10.3389/fpsyg.2026.1977600)
Supplement: Supplementary file 1 [file Data_Sheet_1.PDF]

Supplementary

Supplementary Table S1

Sample Characteristics (N=462)

Characteristic	Category	n	%
**Gender**	Male	214	46.3
	Female	241	52.2
	Prefer not to say	7	1.5
**Age**	21–30	149	32.3
	31–40	187	40.5
	41–50	92	19.9
	51+	34	7.4
**Education**	Diploma/College	69	14.9
	Bachelor	265	57.4
	Master	114	24.7
	Doctorate	14	3.0
**Organizational tenure**	6–12 months	38	8.2
	1–3 years	150	32.5
	4–6 years	119	25.8
	7–10 years	86	18.6
	More than 10 years	69	14.9
**Position**	Non-managerial	293	63.4
	Front-line supervisor	84	18.2
	Middle management	69	14.9
	Senior management	16	3.5
**Weekly working hours**	≤40	157	34.0
	41–45	184	39.8
	46–50	79	17.1
	>50	42	9.1
**Work mode**	On-site	168	36.4
	Hybrid	199	43.1
	Remote	95	20.6

Note. *N* = 462. Each respondent was drawn from a different organization. Percentages may not sum to 100% due to rounding.

Supplementary Table S2

Higher-Order Construct Assessment

Higher-order construct	Lower-order dimension	Loading	α	rho_A	CR	AVE
Digital Autonomy	Decision-Making Autonomy	.931	.846	.846	.928	.866
	Work Methods Autonomy	.931				
Technostrain	Anxiety	.883	.889	.890	.923	.751
	Fatigue	.891				
	Scepticism	.830				
	Inefficacy	.862				
Employee Well-Being	Life Well-Being	.890	.887	.887	.930	.815
	Workplace Well-Being	.913				
	Psychological Well-Being	.906				

Note. Higher-order constructs were assessed using the two-stage approach. Loadings represent the relationships between the lower-order dimensions and their corresponding higher-order constructs. α = Cronbach's alpha; ρA = Dijkstra–Henseler's rho; CR = composite reliability; AVE = average variance extracted.

Supplementary Table S3

Explanatory Power of the Structural Model

Endogenous construct	R^2	Adjusted R^2
Digital Autonomy	.193	.188
Technostrain	.193	.187
Employee Well-Being	.304	.298

Note. R^2 represents the proportion of variance in each endogenous construct explained by its predictors, whereas adjusted R^2 accounts for model complexity by considering the number of predictors. Values are based on the main PLS-SEM structural model.

Supplementary Table S4

Simple Slope Analysis

Outcome	AAE level	Conditional effect of DWF	95% Bootstrap CI
Digital Autonomy	Low (−1 SD)	.523	[.406, .634]
	Mean	.400	[.318, .482]
	High (+1 SD)	.277	[.163, .396]
Technostrain	Low (−1 SD)	.185	[.068, .301]
	Mean	.316	[.233, .398]
	High (+1 SD)	.448	[.326, .565]

Note. Simple slopes were estimated at low (−1 SD), mean, and high (+1 SD) levels of After-hours Availability Expectations (AAE). Confidence intervals are based on 10,000 bootstrap resamples. DWF = Digital Work Flexibility.

Supplementary Table S5

Predictive Assessment of Employee Well-Being

Outcome dimension	No. of indicators	Q^2 _predict range	Mean Q^2 _predict	Indicators with PLS RMSE < LM RMSE
Life Well-Being	6	.109–.151	.128	3/6
Workplace Well-Being	6	.142–.228	.191	3/6
Psychological Well-Being	6	.141–.179	.160	3/6
Overall	18	.109–.228	.160	9/18

Note. Q^2 _predict>0 indicates predictive performance relative to the naïve mean-based benchmark. RMSE comparisons use the linear-model (LM) benchmark.

Supplementary Table S6

Robustness Check Using Unit-Weighted Composite Scores

Panel A

Structural Relationships

Structural relationship	PLS-SEM β	Composite-score OLS β
DWF → Digital Autonomy	.400	.400
DWF × AAE → Digital Autonomy	−.123	−.123
DWF → Technostrain	.316	.316
DWF × AAE → Technostrain	.131	.131
Digital Autonomy → EWB	.346	.346
Technostrain → EWB	−.425	−.425
DWF → EWB	−.047	−.047

Panel B

Net Conditional Indirect Relationships

AAE level	PLS net indirect	OLS net indirect
Low	.102	.102
Mean	.004	.004
High	−.094	−.093

Note. PLS-SEM estimates are compared with OLS estimates based on standardized unit-weighted composite scores. This robustness analysis examines whether the substantive conclusions are sensitive to alternative construct-score weighting approaches. Similar estimates are expected because the alternative composite scores closely approximate the PLS-based construct scores. The results are interpreted as a sensitivity check rather than as a replacement for the main PLS-SEM analysis. DWF = Digital Work Flexibility; AAE = After-hours Availability Expectations; EWB = Employee Well-Being.

Supplementary Table S7

Robustness Check With Control Variables

Structural relationship	Main model β	Model with controls β	Significance with controls
DWF → Digital Autonomy	.400	.409	(p<.001)
DWF → Technostrain	.316	.307	(p<.001)
Digital Autonomy → EWB	.346	.355	(p<.001)
Technostrain → EWB	−.425	−.427	(p<.001)
DWF × AAE → Digital Autonomy	−.123	−.121	(p=.006)
DWF × AAE → Technostrain	.131	.131	(p=.003)
DWF → EWB	−.047	−.057	(p=.217)

Note. The robustness model controlled for gender, age, organizational tenure, managerial position, and weekly working hours.

Supplementary Table S8

Dimension-Level Sensitivity Analysis of Employee Well-Being

Predictor	Life Well-Being	Workplace Well-Being	Psychological Well-Being
Digital Work Flexibility	−.038 n.s.	−.015 n.s.	−.073 n.s.
Digital Autonomy	.264***	.340***	.334***
Technostrain	−.365***	−.413***	−.373***
R^2	.205	.287	.258

Note. ***p<.001; n.s.=nonsignificant.

Supplementary Table S9

Marker-Variable Robustness Assessment

Structural relationship	Main model β	Marker-adjusted β	Δβ
DWF → Digital Autonomy	.400	.399	−.001
DWF×AAE → Digital Autonomy	−.123	−.122	.001
DWF → Technostrain	.316	.318	.002
DWF×AAE → Technostrain	.131	.130	−.001
DWF → EWB	−.047	−.047	.000
Digital Autonomy → EWB	.346	.346	.000
Technostrain → EWB	−.425	−.424	.001

Note. Marker-adjusted coefficients include the marker variable as an additional control. Δβ = marker-adjusted β − main-model β. DWF = Digital Work Flexibility; AAE = After-hours Availability Expectations; EWB = Employee Well-Being.

Supplementary Table S10

Measurement Items and Sources (Adapted English Wording)

Construct / Source	Dimension	Code	Adapted item wording
Digital Work Flexibility (Jones et al. (2008))	Temporal/Spatial Flexibility	DWF1	Digital technologies give me flexibility in deciding when I do my work.
	Temporal/Spatial Flexibility	DWF2	Digital technologies give me flexibility in deciding where I do my work.
Digital Autonomy (Morgeson & Humphrey (2006))	Decision-Making Autonomy	DA-DM1	In digitally mediated work, I have room to use my own initiative and judgment.
	Decision-Making Autonomy	DA-DM2	I can make many work-related decisions on my own.
	Decision-Making Autonomy	DA-DM3	I have substantial autonomy when making work-related decisions.
	Work Methods Autonomy	DA-WM1	I can decide which methods to use to complete my work.
	Work Methods Autonomy	DA-WM2	I have considerable independence in deciding how to do my work.
	Work Methods Autonomy	DA-WM3	I can decide for myself how to go about carrying out my work.
Technostrain (Salanova et al. (2013))	Anxiety	TS-A1	When I use digital technologies for work, I feel tense.
	Anxiety	TS-A2	When I use digital technologies for work, I feel anxious.
	Anxiety	TS-A3	When I use digital technologies for work, I feel uneasy.
	Anxiety	TS-A4	When I use digital technologies for work, I worry about making mistakes.
	Fatigue	TS-F1	Using digital technologies for work leaves me mentally exhausted.
	Fatigue	TS-F2	Using digital technologies for work makes me feel tired.
	Fatigue	TS-F3	I feel drained after sustained use of digital technologies for work.
	Fatigue	TS-F4	After intensive digital work, I need time to recover.
	Scepticism	TS-S1	I have become less interested in using digital technologies for work.
	Scepticism	TS-S2	I sometimes feel detached from the digital technologies I use at work.

Construct / Source	Dimension	Code	Adapted item wording
	Scepticism	TS-S3	I question whether using these technologies at work is worthwhile.
	Scepticism	TS-S4	I feel increasingly cynical about the role of digital technology in my work.
	Inefficacy	TS-I1	I sometimes doubt my ability to use work-related digital technologies effectively.
	Inefficacy	TS-I2	I feel less competent when dealing with digital tools at work.
	Inefficacy	TS-I3	I have difficulty solving problems that arise when I use digital technologies for work.
	Inefficacy	TS-I4	I sometimes feel that I am not as effective as I should be when working with digital technologies.
After-hours Availability Expectations (Derks et al. (2015))	Supervisor Expectations	AAE1	My supervisor expects me to respond to work-related messages during my free time.
	Supervisor Expectations	AAE2	My supervisor expects me to remain reachable for work outside normal working hours.
	Supervisor Expectations	AAE3	My supervisor expects prompt responses to work matters even during leisure time.
	Supervisor Expectations	AAE4	My supervisor would disapprove if I did not respond to work-related messages after hours.
Employee Well-Being (Zheng et al. (2015))	Life Well-Being	LWB1	I am satisfied with my life overall.
	Life Well-Being	LWB2	Most aspects of my life are close to what I would ideally want.
	Life Well-Being	LWB3	I often experience genuine happiness in my life.
	Life Well-Being	LWB4	I feel that my current life circumstances are good.
	Life Well-Being	LWB5	I find my life enjoyable.
	Life Well-Being	LWB6	If I could live my life again, I would make few major changes.
	Workplace Well-Being	WWB1	I am satisfied with the responsibilities I have at work.
	Workplace Well-Being	WWB2	Overall, I am satisfied with my current job.
	Workplace Well-Being	WWB3	I genuinely enjoy the work I do.
	Workplace Well-Being	WWB4	I can find ways to make my work richer and more engaging.
	Workplace Well-Being	WWB5	My work feels meaningful to me.
	Workplace Well-Being	WWB6	I am satisfied with what I have accomplished in my current job.
	Psychological Well-Being	PWB1	I feel that I continue to grow as a person.

Construct / Source	Dimension	Code	Adapted item wording
	Psychological Well-Being	PWB2	I manage the demands of everyday life effectively.
	Psychological Well-Being	PWB3	I generally feel positive and confident about myself.
	Psychological Well-Being	PWB4	I am willing to give my time and help to other people.
	Psychological Well-Being	PWB5	I am good at arranging my work time flexibly.
	Psychological Well-Being	PWB6	I value deep conversations with family and friends that improve mutual understanding.
Marker Variable (Miller & Simmering (2023) approach)	Attitude Toward the Color Blue	ATCB1	I prefer blue to other colors.
	Attitude Toward the Color Blue	ATCB2	I like the color blue.
	Attitude Toward the Color Blue	ATCB3	I like blue clothes.

Note. The wording is adapted for reporting in the present manuscript and is not a verbatim reproduction of the source scales. DWF, Digital Autonomy, AAE, EWB, and ATCB use seven-point agreement scales (1 = strongly disagree, 7 = strongly agree). Technostrain uses a seven-point frequency scale (1 = never, 7 = always or almost every day). The 46 focal items comprise DWF (2), Digital Autonomy (6), Technostrain (16), AAE (4), and EWB (18); the three ATCB items are used only as a marker measure.
