# Supplementary material for: When flexibility feels like control: competing autonomy and Technostrain pathways linking digital work flexibility to employee well-being
Source: Front Psychol. 2026 Oct 22;17:1977600. doi: 10.3389/fpsyg.2026.1977600 (PMC13735578; doi:10.3389/fpsyg.2026.1977600)
Supplement: Supplementary file 2 [file Data_Sheet_2.PDF]

Supplementary Material

Supplementary Table S1

Sample Characteristics (N=462)

Characteristic	Category	n	%
Gender	Male	214	46.3
	Female	241	52.2
	Prefer not to say	7	1.5
Age	21–30	149	32.3
	31–40	187	40.5
	41–50	92	19.9
	51+	34	7.4
Education	Diploma/College	69	14.9
	Bachelor	265	57.4
	Master	114	24.7
	Doctorate	14	3.0
Organizational tenure	6–12 months	38	8.2
	1–3 years	150	32.5
	4–6 years	119	25.8
	7–10 years	86	18.6
	More than 10 years	69	14.9
Position	Non-managerial	293	63.4
	Front-line supervisor	84	18.2
	Middle management	69	14.9
	Senior management	16	3.5
Weekly working hours	≤40	157	34.0
	41–45	184	39.8
	46–50	79	17.1
	>50	42	9.1
Work mode	On-site	168	36.4
	Hybrid	199	43.1
	Remote	95	20.6

Note. N = 462. Each retained respondent was drawn from a different organization under the one-respondent-per-organization recruitment and screening rule. Percentages may not sum to 100% due to rounding.

Supplementary Table S2

Higher-Order Construct Assessment

Higher-order construct	Lower-order dimension	Loading	α	rho_A	CR	AVE
Digital Autonomy	Decision-Making Autonomy	.931	.846	.846	.928	.866
	Work Methods Autonomy	.931				
Technostrain	Anxiety	.883	.889	.890	.923	.751
	Fatigue	.891				
	Scepticism	.830				
	Inefficacy	.862				
Employee Well-Being	Life Well-Being	.890	.887	.887	.930	.815
	Workplace Well-Being	.913				
	Psychological Well-Being	.906				

Note. Higher-order constructs were assessed using the two-stage approach. Loadings represent the relationships between the lower-order dimensions and their corresponding higher-order constructs. α = Cronbach's alpha; rho_A = Dijkstra–Henseler's rho; CR = composite reliability; AVE = average variance extracted.

Supplementary Table S3

Explanatory Power of the Structural Model

Endogenous construct	R^2	Adjusted R^2
Digital Autonomy	.193	.188
Technostrain	.193	.187
Employee Well-Being	.304	.298

Note. R^2 represents the proportion of variance in each endogenous construct explained by its predictors, whereas adjusted R^2 accounts for model complexity.

Supplementary Table S4

Simple Slope Analysis

Outcome	AAE level	Conditional effect of DWF	95% Bootstrap CI
Digital Autonomy	Low (−1 SD)	.523	[.406, .634]
	Mean	.400	[.318, .482]
	High (+1 SD)	.277	[.163, .396]
Technostrain	Low (−1 SD)	.185	[.068, .301]
	Mean	.316	[.233, .398]
	High (+1 SD)	.448	[.326, .565]

Note. Simple slopes were estimated at low (−1 SD), mean, and high (+1 SD) levels of After-hours Availability Expectations (AAE). Confidence intervals are based on 10,000 bootstrap resamples. DWF = Digital Work Flexibility.

Supplementary Table S5

Additional Conditional Total-Association and Direct-Moderation Analyses

Panel A

Conditional Total Associations from the Specified Model

AAE level	Direct DWF→EWB	Net indirect association	Conditional total association	95% bootstrap CI
Low (−1 SD)	−.047	.102	.055	[−.047, .160]
Mean	−.047	.004	−.043	[−.135, .051]
High (+1 SD)	−.047	−.094	−.141	[−.242, −.035]

Panel B

Exploratory Moderation of the Direct DWF–EWB Association

Exploratory interaction	β	95% bootstrap CI	Interpretation
DWF × AAE → EWB	−.082	[−.160, −.000]	Exploratory sensitivity analysis

Panel C

Sensitivity Analysis: Conditional Total Associations When Direct-Path Moderation Is Added

AAE level	Conditional direct association	Expanded-model total association	95% bootstrap CI	Interpretation
Low (−1 SD)	.027	.126	[−.000, .246]	Sensitivity analysis
Mean	−.055	−.051	[−.144, .044]	Sensitivity analysis
High (+1 SD)	−.136	−.228	[−.363, −.084]	Sensitivity analysis

Note. Panel A reports conditional total associations from the specified model, in which the direct DWF→EWB path is not moderated. Panel B reports the exploratory DWF×AAE→EWB interaction added only as a sensitivity analysis. Panel C recomputes conditional total associations in that expanded model. Confidence intervals are based on 10,000 bootstrap resamples. DWF = Digital Work Flexibility; AAE = After-hours Availability Expectations; EWB = Employee Well-Being.

Supplementary Table S6

Robustness Check Using Unit-Weighted Composite Scores

Panel A

Structural Relationships

Structural relationship	PLS-SEM β	Composite-score OLS β
DWF → Digital Autonomy	.400	.400
DWF × AAE → Digital Autonomy	−.123	−.123
DWF → Technostrain	.316	.316
DWF × AAE → Technostrain	.131	.131
Digital Autonomy → EWB	.346	.346
Technostrain → EWB	−.425	−.425
DWF → EWB	−.047	−.047

Panel B

Net Conditional Indirect Relationships

AAE level	PLS net indirect	OLS net indirect
Low	.102	.102
Mean	.004	.004
High	−.094	−.093

Note. PLS-SEM estimates are compared with OLS estimates based on standardized unit-weighted composite scores. DWF = Digital Work Flexibility; AAE = After-hours Availability Expectations; EWB = Employee Well-Being.

Supplementary Table S7

Robustness Check With Control Variables

Structural relationship	Main model β	Model with controls β	Significance with controls
DWF → Digital Autonomy	.400	.409	p<.001
DWF → Technostrain	.316	.307	p<.001
Digital Autonomy → EWB	.346	.355	p<.001
Technostrain → EWB	−.425	−.427	p<.001
DWF × AAE → Digital Autonomy	−.123	−.121	p=.006
DWF × AAE → Technostrain	.131	.131	p=.003
DWF → EWB	−.047	−.057	p=.217

Note. The robustness model controlled for gender, age, organizational tenure, managerial position, and weekly working hours.

Supplementary Table S8

Dimension-Level Sensitivity Analysis of Employee Well-Being

Predictor	Life Well-Being	Workplace Well-Being	Psychological Well-Being
Digital Work Flexibility	−.038 n.s.	−.015 n.s.	−.073 n.s.
Digital Autonomy	.264***	.340***	.334***
Technostrain	−.365***	−.413***	−.373***
R^2	.205	.287	.258

Note. ***p<.001; n.s. = nonsignificant.

Supplementary Table S9

Marker-Variable Robustness Assessment

Structural relationship	Main model β	Marker-adjusted β	Δβ
DWF → Digital Autonomy	.400	.399	−.001
DWF×AAE → Digital Autonomy	−.123	−.122	.001
DWF → Technostrain	.316	.318	.002
DWF×AAE → Technostrain	.131	.130	−.001
DWF → EWB	−.047	−.047	.000
Digital Autonomy → EWB	.346	.346	.000
Technostrain → EWB	−.425	−.424	.001

Note. Marker-adjusted coefficients include the marker variable as an additional control. Δβ = marker-adjusted β − main-model β.

Supplementary Table S10

Measurement Items, Sources, Adaptation Information, and Chinese Wording

Construct	Source	Dimension	Code	Adapted English wording	Chinese wording
Digital Work Flexibility	Jones et al. (2008)	Temporal/Spatial Flexibility	DWF1	Digital technologies give me flexibility in deciding when I do my work.	数字技术使我能够灵活决定何时开展工作。
		Temporal/Spatial Flexibility	DWF2	Digital technologies give me flexibility in deciding where I do my work.	数字技术使我能够灵活决定在哪里开展工作。
Digital Autonomy	Morgeson & Humphrey (2006)	Decision-Making Autonomy	DA-DM1	In digitally mediated work, I have room to use my own initiative and judgment.	在数字化支持的工作中，我有空间运用自己的主动性和判断。
		Decision-Making Autonomy	DA-DM2	I can make many work-related decisions on my own.	我可以自行做出许多与工作有关的决定。
		Decision-Making Autonomy	DA-DM3	I have substantial autonomy when making work-related decisions.	在做出与工作有关的决定时，我拥有较大的自主权。
		Work Methods Autonomy	DA-WM1	I can decide which methods to use to complete my work.	我可以决定采用哪些方法来完成工作。
		Work Methods Autonomy	DA-WM2	I have considerable independence in deciding how to do my work.	在决定如何开展工作时，我具有较大的独立性。
		Work Methods Autonomy	DA-WM3	I can decide for myself how to go about carrying out my work.	我可以自行决定如何开展并完成工作。
Technostrain	Salanova et al. (2013)	Anxiety	TS-A1	When I use digital technologies for work, I feel tense.	当我使用数字技术工作时，我感到紧张。

Construct	Source	Dimension	Code	Adapted English wording	Chinese wording
		Anxiety	TS-A2	When I use digital technologies for work, I feel anxious.	当我使用数字技术工作时，我感到焦虑。
		Anxiety	TS-A3	When I use digital technologies for work, I feel uneasy.	当我使用数字技术工作时，我感到不安。
		Anxiety	TS-A4	When I use digital technologies for work, I worry about making mistakes.	当我使用数字技术工作时，我会担心出错。
		Fatigue	TS-F1	Using digital technologies for work leaves me mentally exhausted.	使用数字技术工作会让我感到精神疲惫。
		Fatigue	TS-F2	Using digital technologies for work makes me feel tired.	使用数字技术工作会让我感到疲劳。
		Fatigue	TS-F3	I feel drained after sustained use of digital technologies for work.	持续使用数字技术工作后，我会感到精疲力竭。
		Fatigue	TS-F4	After intensive digital work, I need time to recover.	在高强度的数字化工作后，我需要时间恢复。
		Scepticism	TS-S1	I have become less interested in using digital technologies for work.	我对使用数字技术开展工作的兴趣有所降低。
		Scepticism	TS-S2	I sometimes feel detached from the digital technologies I use at work.	我有时会对工作中使用的数字技术产生疏离感。
		Scepticism	TS-S3	I question whether using these technologies at work is worthwhile.	我会质疑在工作中使用这些技术是否值得。
		Scepticism	TS-S4	I feel increasingly cynical about the role of digital technology in my work.	我对数字技术在我工作中的作用越来越持怀疑和消极的态度。
		Inefficacy	TS-I1	I sometimes doubt my ability to use work-related digital technologies effectively.	我有时会怀疑自己是否能够有效使用与工作有关的数字技术。
		Inefficacy	TS-I2	I feel less competent when dealing with digital tools at work.	在应对工作中的数字工具时，我会觉得自己不够胜任。
		Inefficacy	TS-I3	I have difficulty solving problems that arise when I use digital technologies for work.	当我使用数字技术工作时，如果出现问题，我会觉得难以解决。
		Inefficacy	TS-I4	I sometimes feel that I am not as effective as I should be when working with digital technologies.	使用数字技术工作时，我有时会觉得自己的工作效率没有达到应有水平。
After-hours Availability Expectations	Derks et al. (2015)	Supervisor Expectations	AAE1	My supervisor expects me to respond to work-related messages during my free time.	我的直接上级希望我在空闲时间回复与工作有关的消息。
		Supervisor Expectations	AAE2	My supervisor expects me to remain reachable for work outside normal working hours.	我的直接上级希望我在正常工作时间之外仍保持工作联系。
		Supervisor Expectations	AAE3	My supervisor expects prompt responses to work matters even during leisure time.	即使在休闲时间，我的直接上级也希望我及时回应工作事务。
		Supervisor Expectations	AAE4	My supervisor would disapprove if I did not respond to work-related messages after hours.	如果我在下班后没有回复与工作有关的消息，我的直接上级会表示不满。
Employee Well-Being	Zheng et al. (2015)	Life Well-Being	LWB1	I am satisfied with my life overall.	总体而言，我对自己的生活感到满意。
		Life Well-Being	LWB2	Most aspects of my life are close to what I would ideally want.	我生活中的大多数方面都接近我理想中的状态。
		Life Well-Being	LWB3	I often experience genuine happiness in my life.	我经常在生活中体验到真正

Construct	Source	Dimension	Code	Adapted English wording	Chinese wording
					的快乐。
		Life Well-Being	LWB4	I feel that my current life circumstances are good.	我觉得自己目前的生活状况很好。
		Life Well-Being	LWB5	I find my life enjoyable.	我觉得自己的生活很愉快。
		Life Well-Being	LWB6	If I could live my life again, I would make few major changes.	如果我可以重新生活一次，我不会对现在的生活做出太多重大改变。
		Workplace Well-Being	WWB1	I am satisfied with the responsibilities I have at work.	我对自己承担的工作职责感到满意。
		Workplace Well-Being	WWB2	Overall, I am satisfied with my current job.	总体而言，我对目前的工作感到满意。
		Workplace Well-Being	WWB3	I genuinely enjoy the work I do.	我真正享受自己所做的工作。
		Workplace Well-Being	WWB4	I can find ways to make my work richer and more engaging.	我能找到让自己的工作更加丰富、更具投入感的方法。
		Workplace Well-Being	WWB5	My work feels meaningful to me.	我的工作对我来说很有意义。
		Workplace Well-Being	WWB6	I am satisfied with what I have accomplished in my current job.	我对自己在当前工作中取得的成就感到满意。
		Psychological Well-Being	PWB1	I feel that I continue to grow as a person.	我觉得自己作为一个人仍在不断成长。
		Psychological Well-Being	PWB2	I manage the demands of everyday life effectively.	我能够有效应对日常生活中的各种要求。
		Psychological Well-Being	PWB3	I generally feel positive and confident about myself.	总体而言，我对自己持积极态度并且有信心。
		Psychological Well-Being	PWB4	I am willing to give my time and help to other people.	我愿意付出自己的时间并帮助他人。
		Psychological Well-Being	PWB5	I am good at arranging my work time flexibly.	我善于灵活安排自己的工作时间。
		Psychological Well-Being	PWB6	I value deep conversations with family and friends that improve mutual understanding.	我重视与家人和朋友进行深入交谈，以增进彼此理解。
Marker Variable	Miller & Simmering (2023) approach	Attitude Toward the Color Blue	ATCB1	I prefer blue to other colors.	与其他颜色相比，我更喜欢蓝色。
		Attitude Toward the Color Blue	ATCB2	I like the color blue.	我喜欢蓝色。
		Attitude Toward the Color Blue	ATCB3	I like blue clothes.	我喜欢蓝色的衣服。

Note. The English wording shown is the adapted wording used in this study; the Chinese column reports the corresponding questionnaire wording. Jones et al. (2008) reports the two DWF items by content—control/flexibility over scheduling when and where one worked—rather than reproducing full questionnaire stems. The present DWF items preserve those temporal and spatial domains in digitally enabled work. Derks et al. (2015) reports a four-item supervisor-expectations measure regarding smartphone use and after-work connectivity but provides example rather than all four item stems; accordingly, the four AAE items are presented here as adapted, not verbatim, wording, with the supervisor-focused after-hours referent preserved. All adaptations were included in back-translation and expert review. DWF, Digital Autonomy, AAE, EWB, and ATCB use seven-point agreement scales (1 = strongly disagree, 7 = strongly agree); Technostrain uses a seven-point frequency scale (1 = never, 7 = always or almost every day).

Supplementary Table S11

Recruitment and Data Screening Summary

Stage / criterion	Excluded at stage	Remaining N	Comment
Invited employees	—	818	Initial invitations
Returned questionnaires	—	539	Response rate = 65.9%
Eligibility criteria not met	14	525	Sequential first-trigger screening
Substantial missing key data	8	517	Sequential first-trigger screening
Unusually short completion time	17	500	Sequential first-trigger screening
Straight-lining / insufficient response variation	13	487	Sequential first-trigger screening
Failed attention checks	16	471	Sequential first-trigger screening
Duplicate or otherwise non-independent submissions	9	462	Organization-linked records + SOJUMP duplicate/device screening
Final analytic sample	—	462	85.7% of returned questionnaires; 56.5% of invitations; one retained employee per organization

Note. Screening criteria were applied sequentially and mutually exclusively, with each questionnaire assigned to the first criterion triggered. Organization-linked recruitment records and SOJUMP duplicate/device screening were used to identify potentially non-independent submissions; when multiple eligible submissions were linked to the same organization, only the first complete eligible response was retained. The six exclusion counts sum to 77.
